# Filamentation Profiling Reveals Multiple Transcription Regulators Contributing to the Differences Between *Candida albicans* and *Candida dubliniensis*

**DOI:** 10.1111/mmi.70012

**Published:** 2025-07-17

**Authors:** Teresa Meza‐Davalos, Luis F. García‐Ortega, Eugenio Mancera

**Affiliations:** ^1^ Departamento de Ingeniería Genética Unidad Irapuato, Centro de Investigación y de Estudios Avanzados del Instituto Politécnico Nacional Irapuato Mexico

**Keywords:** *Candida albicans*, *Candida dubliniensis*, filamentation, pathogenic fungi, transcription regulator

## Abstract

*Candida dubliniensis* is the most closely related species to *C. albicans* , one of the leading causes of fungal infections in humans. However, despite sharing many characteristics, *C. dubliniensis* is significantly less pathogenic. To better understand the molecular underpinnings of these dissimilarities, we focused on the regulation of filamentation, a developmental trait fundamental for host colonization. We generated a collection of 44 *C. dubliniensis* null mutants of transcription regulators whose orthologs in *C. albicans* had been previously implicated in filamentous growth. These regulators are very similar at the sequence level, but phenotypic screening identified several mutants with contrasting interspecific filamentation phenotypes beyond previously known differences. Bcr1, a well‐known regulator of biofilm formation, stands out as its mutant mainly showed a filamentation defect in *C. dubliniensis*. Phenotypic and transcriptional characterization showed that the *bcr1* defect is condition dependent and that this regulator plays a central role in the filamentation of *C. dubliniensis*, possibly by regulating the hyphal activator Ume6. Overall, our results suggest that several regulatory pathways are involved in the filamentation differences between *C. albicans* and *C. dubliniensis* and show that the *C. dubliniensis* mutant collection is a valuable resource to compare, at a molecular level, these species of medical relevance.

## Graphical abstract

*Candida albicans* is a major fungal pathogen whose virulence is linked to filamentation. Its close relative, *C. dubliniensis*, filaments less efficiently and causes disease more rarely. To explore regulatory differences, we created *C. dubliniensis* mutants lacking transcription regulator orthologs that have been associated with filamentation in *C. albicans* . This revealed key regulators, including Bcr1, whose function has diverged. Our study highlights molecular differences in filamentation and provides tools to further investigate *Candida* pathogenicity.

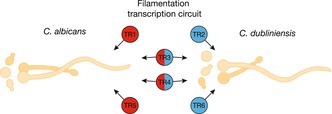

## 1 Introduction

Fungi from the genus *Candida* are among the most important human pathogens (Katsipoulaki et al. 2024; Parambath et al. 2024). They are capable of causing a spectrum of diseases, ranging from mild superficial infections of the oral cavity and vagina to severe, life‐threatening systemic conditions with high levels of morbidity and mortality (Pappas et al. 2018; Vila et al. 2020). These infections pose a significant risk especially to individuals with compromised immune systems (Katsipoulaki et al. 2024; Parambath et al. 2024). However, these fungi are also found as part of the microbial communities that commensally inhabit our bodies and therefore are considered opportunistic pathogens (Kondori et al. 2020; Rao et al. 2021; Yan et al. 2024).

Most medically relevant *Candida* species belong to the CTG (CUG‐Ser1) clade, a monophyletic group of ascomycetous yeasts characterized by the translation of the CUG codon as serine instead of leucine (Butler et al. 2009). Within this group, *Candida albicans* stands out as the most virulent species, being a leading cause of both superficial and systemic infections in humans (Parambath et al. 2024). The clade includes other important opportunistic pathogens, but also many species that have not been associated with humans or that are much rarer etiological agents (Gabaldon et al. 2016; Opulente et al. 2024). This is the case with *C. dubliniensis*, the species most closely related to *C. albicans* phylogenetically, yet considerably less prevalent in clinical settings. For example, although regional differences have been observed, a previous multi‐country surveillance study estimated that *C. albicans* was responsible for approximately 65% of the infections caused by *Candida* species, while *C. dubliniensis* accounted for fewer than 0.1% (Pfaller et al. 2010; Turner and Butler 2014). Moreover, the World Health Organization has recently included *C. albicans* in the critical priority group among fungal pathogens, while *C. dubliniensis* has not been considered at all (Rodrigues and Nosanchuk 2023). In agreement, *C. dubliniensis* has been shown to be less virulent in several murine models of infection (Gilfillan et al. 1998; Stokes et al. 2007; Vilela et al. 2002). Given their virulence differences but evolutionary proximity—the two species are estimated to have diverged 20 million years ago (Moran et al. 2011; Moran et al. 2012)–*C. dubliniensis* has been a useful comparative model to understand the underpinnings of *C. albicans* pathogenicity (Jackson et al. 2009).

In *C. albicans* , the morphological transition between yeast and filamentous cells (hyphae and pseudohyphae) is important for the colonization of the human host and disease causation (Mayer et al. 2013; Sudbery 2011; Wilson et al. 2016). Changes in cell shape have been suggested to allow disruption of host cells and tissue, while the differential expression of virulence factors between yeasts and filaments is also key for adaptation to different environments in the host, including interactions with other microorganisms in these habitats (Liang et al. 2024). The ability to filament is shared by *C. dubliniensis*, as this species is also able to form both hyphae and pseudohyphae. However, relative to *C. albicans* , *C. dubliniensis* has been observed to filament more rarely, and the range of known stimuli that trigger filament formation in this species is much narrower (O'connor et al. 2010; Vilela et al. 2002). Not surprisingly, the decreased ability to transition from yeast to hyphae has been associated with its reduced virulence (Gilfillan et al. 1998; Stokes et al. 2007; Vilela et al. 2002). In agreement, experiments in murine models of infection have shown that *C. dubliniensis* cells in the stomach and kidney remain predominantly in the yeast form, whereas *C. albicans* cells exhibited both yeasts and hyphae (Stokes et al. 2007; Vilela et al. 2002).

Multiple signaling pathways and transcription regulators (TRs) have been found to control the switch between yeast and filamentous forms in *C. albicans* , indicating that the morphological transition is quite complex at a molecular level (Liu 2001; Polvi et al. 2019). Changes in some of these pathways and regulators have been associated with the filamentation differences with *C. dubliniensis*. For example, differential expression of the transcriptional repressor *NRG1* has been shown to be partially responsible for the filamentation differences as it is quickly downregulated in *C. albicans* by several stimuli of the human host, while its expression does not decrease as sharply in *C. dubliniensis* (Moran et al. 2007). Similarly, overexpression of the TR Ume6 that is known to be repressed by Nrg1 (Moran et al. 2007; Sullivan and Moran 2011) has been associated with filamentation of *C. albicans* under several conditions, but in *C. dubliniensis* its expression change requires starvation, one of the few conditions where this species is known to filament (O'connor et al. 2010). Although the genomes of *C. albicans* and *C. dubliniensis* are very similar, their comparison also shed light into the filamentation differences of the two species (Jackson et al. 2009). For instance, key hypha‐specific virulence factors such as Hyr1, Als3, and some members of the secreted aspartyl proteinase (SAP) family are absent in *C. dubliniensis* (Caplice and Moran 2015; O'connor et al. 2010). These differences have also been observed when comparing genome‐wide transcriptional profiles of the two species, revealing hypha‐induced genes in *C. albicans* that do not change their expression in *C. dubliniensis* (Caplice and Moran 2015).

Given the complexity of the regulatory circuit that controls filamentation in *C. albicans* —at least 45 TRs have been associated with this transition—additional differences with *C. dubliniensis* could be expected. To further understand the dissimilarities at a molecular level, we generated a deletion collection of most of the *C. dubliniensis* orthologs of the TRs that have been associated with filamentation in *C. albicans* . Comparative profiling under inducing conditions revealed contrasting filamentation phenotypes in several of the mutants, beyond the previously known differences. Transcriptional profiling of the *bcr1* mutant, one of the regulators with marked differences, showed extensive interchange of target genes. Overall, our work suggests considerable rewiring in the regulatory circuits that control filamentation in these two closely related species with contrasting clinical characteristics.

## 2 Results

### 2.1 Transcription Regulators That Control Filamentation Are Conserved at the Sequence Level Between *C. albicans* and *C. dubliniensis*

**FIGURE 1:**
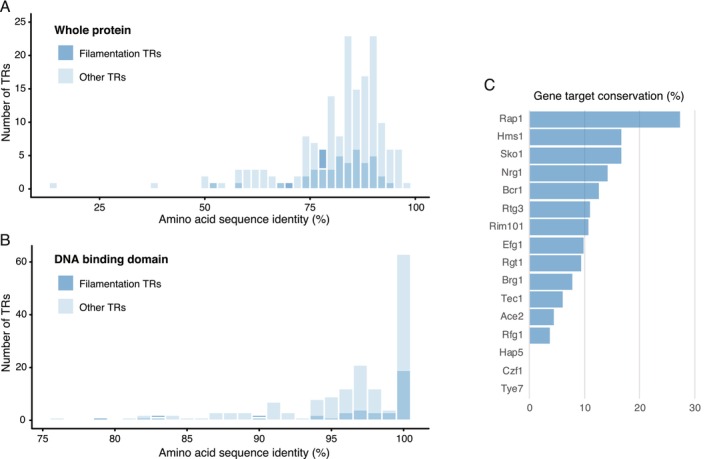
Filamentation TR are conserved at the protein level between *C. albicans* and *C. dubliniensis*, but their putative target genes have diverged considerably. (A) Distribution of the amino acid sequence identity (%) of TR orthologs not involved in filamentation (light blue) and those related to filamentation (dark blue) along the whole protein. Bars in the histograms where the two categories of TRs overlap are shown in an intermediate tone of blue. (B) As (A), but only for the DNA binding domain of the TRs. Only the TRs for which a DNA binding domain has been defined are included in (B). (C) Conservation (%) of computationally predicted target genes between filamentation TR orthologs. Target genes were defined by the presence of the DNA binding sequence motif in the upstream region of a gene (Experimental Procedures). Only TR for which a DNA binding sequence motif has been previously reported in *C. albicans* were included.

To better understand the differences in the molecular mechanisms that control filamentation between *C. albicans* and *C. dubliniensis*, we focused on the TRs that have been associated with this cellular process. TRs function as hubs in the regulation of cellular metabolism and, given the gene deletion collection available for their study in *C. albicans* , they represented an ideal entry point. Considering previously defined TR (Homann et al. 2009), we found 45 TRs whose knockout mutant in *C. albicans* had a phenotype associated with filamentation according to the Candida Genome Database (CGD) (Experimental Procedures; Table S1). These TRs represent between 15% and 20% of the total TRs present in *C. albicans* , and all have a one‐to‐one ortholog in *C. dubliniensis* according to the Candida Gene Order Browser (CGOB) (Maguire et al. 2013). To assess the degree of conservation of the 45 TRs between *C. albicans* and *C. dubliniensis*, we first aligned the protein sequence of each pair of orthologs. The average sequence identity between orthologs is 81.1% (Figure 1A) and, as expected, it is even higher in the DNA binding domain (96.3%, Figure 1B). At the amino acid sequence level, this group of TRs is not atypical since their identity falls well within the range of identity of all the TR ortholog pairs between these two species (Figure 1A). This is also the case for the DNA binding domain (Figure 1B). In both species, the protein domains that are most common among the 45 filamentation TRs are the Zinc finger C2H2‐type domain (IPR013087), the Zn(2)Cys(6) fungal‐type DNA‐binding domain (IPR001138) and the Myc‐type, basic helix–loop–helix (bHLH) domain (IPR011598), while, in the overall set of TRs, the most frequent ones are the Zn(2)Cys(6) fungal‐type DNA‐binding domain (IPR001138), the Zinc finger C2H2‐type (IPR013087) and the Transcription factor domain, fungi (IPR007219). The conservation in protein domain composition in the TRs of *C. albicans* and *C. dubliniensis* is in agreement with the high sequence identity of the TRs between the two species.

### 2.2 The Predicted Target Genes Based on DNA‐Binding Motives of the Filamentation Transcription Regulators Are Very Different Between *C. albicans* and *C. dubliniensis*

Contrary to the high conservation that we observed at the amino acid sequence level, previous experimental determination of the target genes of six of these TRs has shown considerable divergence between *C. albicans* and *C. dubliniensis* (Mancera et al. 2021). To further explore the degree of conservation in target genes, we computationally identified targets based on the presence of the DNA binding motif in their upstream region (Experimental Procedures). The motifs of only 16 TRs have been determined in *C. albicans* , and we assumed that these motifs are conserved in *C. dubliniensis* given the sequence similarity between the ortholog TRs, especially in the DNA binding domain. The degree of conservation that we observed in putative target genes fell within the range of previous comparisons between these two species (Mancera et al. 2021; Nobile et al. 2012). The TR whose putative target genes are most conserved is Rap1, and only 27% of its targets are shared between *C. albicans* and *C. dubliniensis* (Figure 1C). On the other end of the distribution, for three TRs (Czf1, Hap5, and Tye7) there were no putative gene targets shared between the two species (Figure 1C). Overall, our observations suggest that the filamentation differences between *C. albicans* and *C. dubliniensis* could be due to the target gene differences of the TRs that regulate the process. It is important to keep in mind that despite the high sequence similarity between the protein DNA binding domains of the TRs, it is still possible that there are differences in the DNA motifs that these regulators bind. These differences would diminish the predictive power that the presence of the motif has to define a target gene. Independently, our results showed that without further experimentation, it would be difficult to predict the specific TRs that contribute to the filamentation differences between the two species.

### 2.3 A Transcription Regulator Mutant Collection to Identify Differences in the Regulation of Filamentation Between *C. albicans* and *C. dubliniensis*

To experimentally identify differences between *C. albicans* and *C. dubliniensis* in the function of the TRs that are involved in filamentation, we generated a gene knockout collection of the orthologs of the *C. albicans* filamentation TR in *C. dubliniensis*. Of the 45 filamentation TRs in *C. albicans* , a knockout mutant had already been generated in *C. dubliniensis* for five of them (Mancera et al. 2021). A *nrg1* null mutant had also been generated, but in a different genetic background (Moran et al. 2007) and therefore we also constructed the deletion strain. The null mutants for the 40 TRs were generated using the same genetic engineering strategy used to generate the *C. albicans* TR mutants, employing two auxotrophic markers to tandemly delete the two alleles of a given gene (Homann et al. 2009). For each gene, one independent homozygous mutant was generated in two different *C. dubliniensis* parental auxotrophic strains so that the phenotype of the deletion could be assessed in replicates.

Even though several transformation attempts were performed, we could not generate the homozygous *tup1* mutant. This was surprising given that deletion mutants of this gene have been previously reported in *C. albicans* and *C. tropicalis* (Braun and Johnson 1997; Gong et al. 2019). It is possible that this TRs is essential in *C. dubliniensis* or that its gene is located in an aneuploid genomic region, and an extra allele is present in this species. Together with the 5 deletion mutants previously generated, we put together a collection of 44 homozygous and 44 heterozygous knockout mutants to experimentally assess the function of the *C. dubliniensis* orthologs of the filamentation TRs.

### 2.4 Multiple Transcription Regulators Contribute to the Filamentation Differences Between *C. albicans* and *C. dubliniensis*

To test whether the deletion mutants of the TRs have a filamentation defect, we performed filamentation time courses of all the mutants in both species in parallel. As detailed in Experimental Procedures, filamentation was induced by transferring strains to water with 10% fetal bovine serum (FBS) at 37°C since both species are known to form hyphae under these conditions. Morphological changes were monitored under the microscope at the time of transferring to the induction media (“zero” time‐point) and after 1, 3, and 5 h of induction (Figures S1 and S2). Filamentation was quantitatively estimated as the fraction of cells that formed filamentous morphologies at the first three time points and by estimating the Morphological Index (MI) at the 1‐h time point (Merson‐Davies and Odds 1989). To clarify phenotypic inconsistencies between the two isolates of the mutants of five genes (*PHO4*, *GRF10*, *AFT2*, *ADR1*, and *FGR15*), we generated an additional homozygous mutant.

**FIGURE 2:**
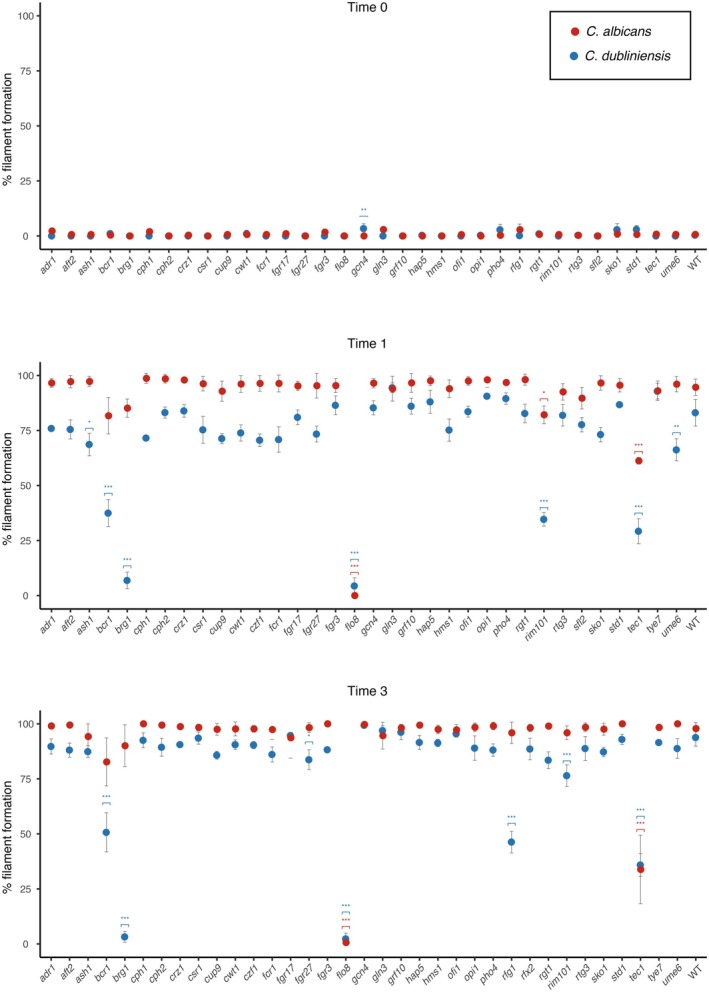
Multiple TR contribute to the filamentation differences between *C. albicans* and *C. dubliniensis*. Comparative phenotypic characterization of the TR mutants in filamentation inducing conditions (water with 10% FBS) through time. The percentage of the cells that showed filamentous morphologies is shown at each time point. Red dots represent the *C. albicans* mutants while blue ones the corresponding *C. dubliniensis* ortholog. Upper panel shows the phenotype when cells were transferred to the inducing conditions (Time 0) and the mid and lower panels show the phenotype after one (Time 1) and three (Time 3) hours after induction, respectively. Errors bars are the standard deviation of three replicates and only one of the isolates of each *C. dubliniensis* mutant is shown. Asterisks denote statistically significant differences between the mutant and the wild type strain (Bonferroni corrected *t*‐test, *p < 0.05, **p < 0.01, ***p < 0.001). Only mutants that could be quantitatively assessed at each time point are included in each of the panels and therefore the mutants in each panel (horizontal axis) are not the same for all three plots.

We could not quantitate the percentage of filamentation in at least one of the time points for 12 TR mutants (*ace2*, *czf1*, *efg1*, *fgr15*, *nrg1*, *rap1*, *rca1*, *rfg1*, *rfx2*, *ssn6*, *stp2*, *and tye7*) given that they showed morphologies that could not be clearly classified as individual yeast cells or filaments in one or the two species (Figure S2). For all of these 12 mutants but *rfg1*, the aberrant morphology was already evident in one of the two species at the time of transferring the cells to the induction media, suggesting that the morphology is independent of the filamentation stimulus. Most of these mutants formed elongated cells or aggregates not observed in the wildtype strains at time point zero. The mutants *ace2*, *fgr15*, *rap1*, *rca1*, *ssn6*, and *stp2* showed deviant morphology in both species and are in agreement with previous reports in *C. albicans* (Braun et al. 2001; Homann et al. 2009; Hwang et al. 2003; Moran et al. 2007; Mulhern et al. 2006; Vylkova and Lorenz 2014). On the other hand, *tye7*, *czf1*, and *rfx2* exhibited filamentous growth in *C. dubliniensis* while maintaining a yeast form in *C. albicans* . Additionally, the mutant *nrg1* displayed hyperfilamentous growth solely in *C. albicans* . The *efg1* mutant did not filament in any of the three time points considered; however, it formed elongated cells, particularly in *C. dubliniensis*. Even when these cells were clearly not filaments, they made quantitatively estimating filamentation difficult. From the mutants that could be quantitatively characterized at time zero (Figure 2A), the *gcn4* mutant showed a statistically significant difference in filamentation (Bonferroni corrected *t*‐test, *p* < 0.05), but only in *C. dubliniensis*. This mutant showed increased filamentation compared to the wildtype strain, although the difference was mild at this time point.

After 1 h under the filamentation inducing conditions, apart from the mutants described above that could not be quantified at the zero‐time point, the *rfg1* mutant also showed a morphology in *C. albicans* that impeded quantification (Figure S2). From the mutants in which filamentation could be quantified (Figure 2B), *flo8*, *rim101*, and *tec1* showed a statistically significant reduction in the number of filamenting cells in both species (Bonferroni corrected *t*‐test, *p* < 0.05). On the other hand, the mutants of *ASH1*, *BCR1*, *BRG1*, and *UME6* showed a statistically significant reduction in filamentation, but only in *C. dubliniensis* (Figure 2B).

At the 3h time‐point in filamentation conditions, the *flo8* and *tec1* mutants continued showing a filamentation defect in both species, but in *rim101* the defect was only statistically significant for *C. dubliniensis* (Figure 2C). The filamentation defect of the knockout strains of *BCR1* and *BRG1* in *C. dubliniensis* persisted at this time point, but *ash1* and *ume6* did not show statistically significant differences anymore. The only mutant that showed a filamentation defect at the 3‐h time‐point that had not shown the phenotype before was *fgr27*, and it did so only in *C. dubliniensis* (Figure 2C). Contrary to the 1‐h time‐point, after 3 h, filamentation could be quantitated in the *rfg1* and *rfx2* mutants, and only the former showed a statistically significant difference compared to the wildtype strain in *C. dubliniensis* (Figure 2C). Overall, among the mutants for which filamentation could be quantitated, there were two main phenotypes: strains that showed more filaments than the wildtype strain at time zero, and mutants that had reduced numbers of filamentous cells after one and 3 h in the inducing conditions (Figure 2).

To provide a quantitative estimation of the morphological changes in the mutants, we also estimated the MI at the 1‐h time point (Experimental Procedures and Table S2). Three mutants showed a statistically significant reduction in MI compared to the wildtype strain in both species (*efg1*, *flo8*, and *stp2*). This agrees with the inability of these three mutants to form filamentous cells. No other mutant showed statistically significant changes in their MI in *C. dubliniensis*, while 12 additional mutants had a significantly reduced MI only in *C. albicans* (*bcr1*, *brg1*, *cph2*, *cup9*, *gln3*, *grf10*, *hms1*, *opi1*, *rca1*, *rim101*, *ssn6*, and *tec1*; Table S2). Overall, these results are in agreement with the deficiencies identified by counting the proportion of filamentous cells—only the defects of *cph2*, *cup9*, *gln3*, *grf10*, *hms1*, and *opi1* had not been identified (Table S2).

In total, including the mutants with a defect in their MI and those for which filamentation was not quantitated (Figure S2), 28 of the TR mutants (63.6%) showed a filamentation phenotype different from that of the wildtype strain in one of the two species at least in one of the time points analyzed. If we only consider *C. albicans* , 17 mutants (38.6%) showed a filamentation defect. Of these mutants, 10 also showed a defect in *C. dubliniensis*, while there were 10 strains that only showed a phenotype in *C. dubliniensis*. It is important to point out that we performed the screen under specific inducing conditions and that alternative filamentation stimuli may be needed to expose the filamentation phenotypes of the *C. albicans* mutants that did not show a filamentation defect in our assay. However, in summary, our results suggest that several TRs contribute to the differences in the way filamentation is regulated in *C. albicans* and *C. dubliniensis*.

### 2.5 The Difference in Filamentation Between the *C. albicans* and *C. dubliniensis bcr1* Mutant Is Condition Dependent

One of the mutants that showed marked differences in filamentation between *C. albicans* and *C. dubliniensis* was *bcr1* (Figure 2). In *C. dubliniensis*, this strain showed a considerable reduction in the number of filamentous cells after 1 h in the inducing conditions, and after 3 and 5 h, it exhibited few filaments that resembled pseudohyphae. On the other hand, the mutant of the ortholog in *C. albicans* showed a similar fraction of filamentous cells to the wildtype strain at the three time points, although it had a reduced MI at the 1‐h time point (Table S2). The phenotype in *C. albicans* is consistent with previous work showing that *BCR1* is not required for hyphal formation (Nobile et al. 2012; Nobile and Mitchell 2005). On the other hand, this gene is known to be required for biofilm formation in both species and in other *Candida* species that are phylogenetically further apart (Mancera et al. 2021).

**FIGURE 3:**
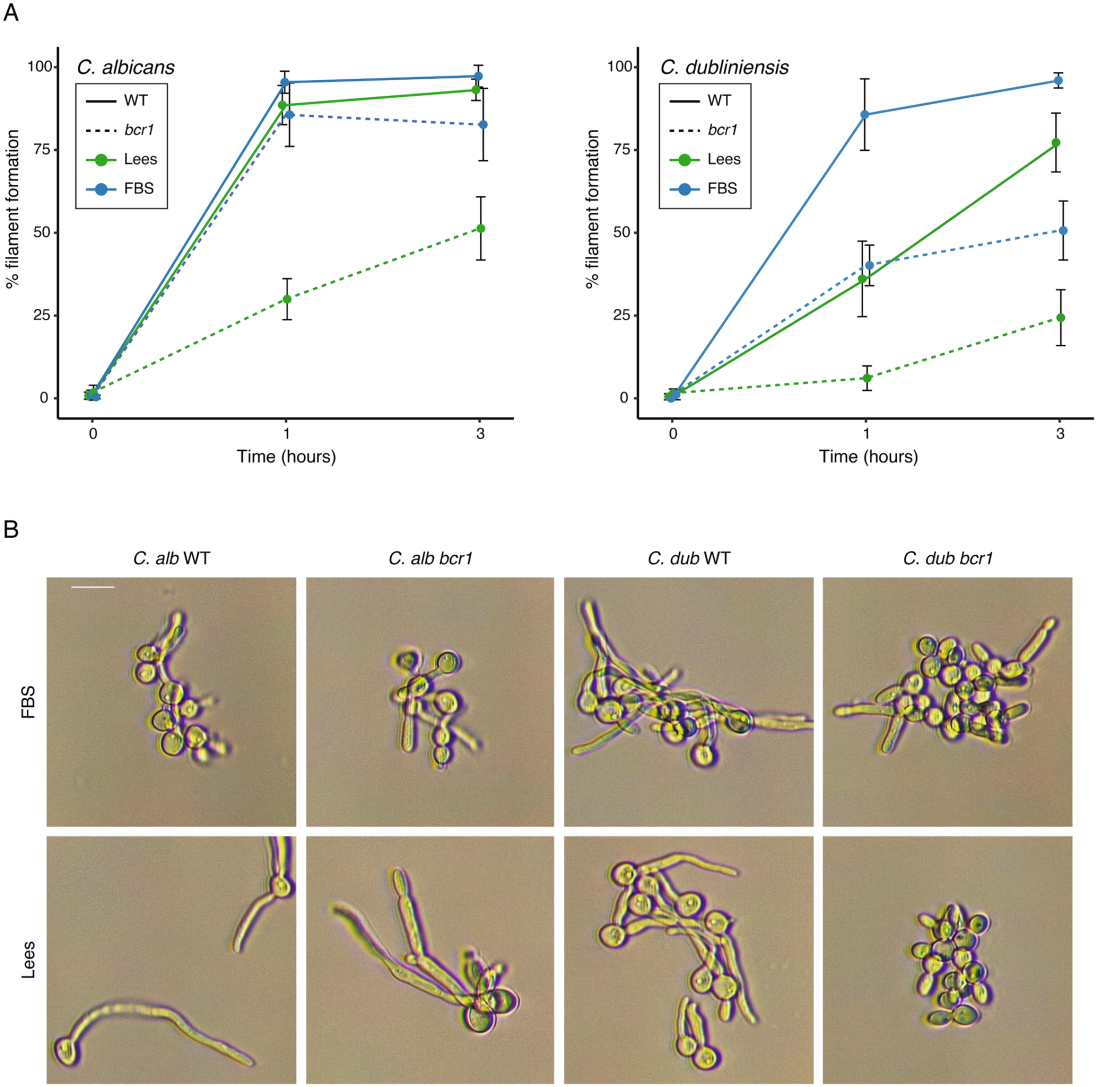
Filamentation differences of the *bcr1* mutant between species are media specific. (A) Quantification of the portion of cells that showed filamentous morphologies through time (0, 1, and 3 h after induction) in the homozygous *bcr*1 mutant (discontinuous lines) compared with the wildtype strain (WT, continuous lines) in two different inducing media, water with 10% FBS (FBS, blue lines) and water with 10% Lee's medium (Lees, green lines). Error bars show the standard deviation of three replicates. (B) Representative micrographs of the wildtype and *bcr1* mutant of the two species (*C. alb* and *C. dub*) filamenting in the same two media (FBS and Lees). Micrographs were taken under an optical microscope after 3 h in the inducing condition and the scale bar represents 20 μm.

To investigate whether the observed differences in the *bcr1* mutants of the two species were specific to the growth conditions employed in the screen, we performed filamentation assays using Lee's medium as a defined basal medium for induction. It has been reported that to induce filamentation in *C. dubliniensis*, a combination of temperature and pH shift in nutrient‐depleted media is required, which can be achieved by transferring cells to Lee's medium at 37°C. In addition, unlike FBS, this medium does not include any animal‐based components (Caplice and Moran 2015; Lee et al. 1975). As can be observed in Figure 3, in contrast to the phenotype in FBS, the *C. albicans bcr1* mutant showed decreased filamentation in Lee's medium after 1 h. After 3 h in this medium, the defect of the *C. albicans* mutant was milder but still statistically significant. *C. dubliniensis* did not filament as efficiently in this medium, but the defect of the *bcr1* mutant was evident along the time points (Figure 3A).

Since the FBS and Lee's media were originally tailored for *C. dubliniensis* filamentation (Caplice and Moran 2015), we then examined the morphology of the *bcr1* mutants in response to other inducing cues that have been previously used for *C. albicans* (Experimental Procedures). Of the three media tested, only SD supplemented with 0.75% glucose and 50% FBS induced filamentation in *C. dubliniensis*, and the *bcr1* mutant did not filament under this condition as was seen with FBS or Lee's medium (Figure S3). The *C. albicans bcr1* mutant did not show a filamentation defect, behaving as the reference strain in the three additional media tested.

We also assessed the role of the change in pH in the filamentation of the mutant, as it has been described as a crucial factor to induce filamentation in these species (Caplice and Moran 2015). For this, we set the pre‐induction culture in Lee's media adjusted at pH 7.2 instead of pH 4.5 as had been done for all previous assays. In this condition, the *C. albicans bcr1* mutant showed an important filamentation defect not seen in the reference strain (Figure S3). This was observed when using FBS as the inducing condition, both in water and YPD medium. When starting at pH 7.5, the *C. dubliniensis bcr1* mutant showed the same defect as when the pre‐induction culture was set at pH 4.5. Interestingly, when the pre‐induction culture was set at pH 7.5 and RPMI medium supplemented with FBS was used for induction, the *C. dubliniensis bcr1* mutant did filament while the reference strain did not (Figure S3). Under this condition, both *C. albicans* strains filamented. Overall, these results showed that the filamentation requirement for Bcr1 depends on the conditions used to induce filamentation. However, it was also clear that *BCR1* is needed for filamentation in more conditions in *C. dubliniensis* than in *C. albicans* .

### 2.6 Considerable Differences in the Gene Expression Program Controlled by Bcr1 Between *C. albicans* and *C. dubliniensis*

To further understand the differences in the regulatory role of Bcr1 between *C. albicans* and *C. dubliniensis*, we focused on the genes that are controlled by this regulator during filamentation. We performed RNA‐seq of the wildtype and the *bcr1* mutant in both species after 1 h of filamentation induction with FBS or Lee's media. Comparison of the transcription profiles of the wildtype and *bcr1* mutant allowed us to identify the genes whose expression depends directly or indirectly on Bcr1 in the two different inducing conditions. Adding the two conditions and including all the genes whose expression change is statistically significant independently of the magnitude of the change, 2198 and 2296 genes were differentially expressed in the *bcr1* mutant in *C. albicans* and *C. dubliniensis*, respectively (Supplementary Table S3). This represents close to one third of the total number of genes in the genomes of these species. Comparing the expression profiles between filamentation conditions, we observed that the fraction of differentially expressed genes that are shared between media is larger in *C. dubliniensis* (31.7%) than in *C. albicans* (19.7%). This is in agreement with the observation that the filamentation defect was observed in both media in the mutant of *C. dubliniensis*, but not in that of *C. albicans* .

**FIGURE 4:**
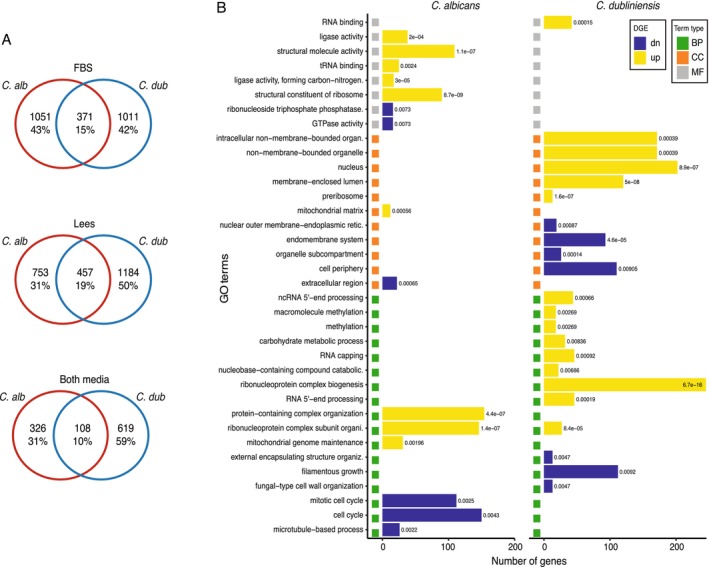
The transcription circuits controlled by Bcr1 have diverged considerably between *C. albicans* and *C. dubliniensis*. (A) Venn diagrams of the overlap between differentially expressed genes in the *bcr1* mutant of *C. albicans* and *C. dubliniensis*. The top diagram shows the overlap when inducing by transferring to water with 10% FBS (FBS), the middle diagram when inducing with water with 10% Lee's medium (Lees), and the bottom diagram for differentially expressed genes in both media. All statistically significant differentially expressed genes were included, independently of the magnitude and direction of the expression change. (B) Comparison of the GO terms enriched among the differentially expressed genes in the two species. Only genes that were differentially expressed in both inducing conditions were included in the analysis. Yellow horizontal bars show categories enriched in upregulated genes while blue bars in downregulated genes. Colored squares at the left of each bar denote the type of GO term: BP, biological process; CC, cellular compartment; MF, molecular function. The numbers to the right of each horizontal bar are the enrichment *p*‐value.

In the *bcr1* mutant, the overlap in differentially expressed genes between the two species was 15.2% in FBS and 19.0% in Lee's medium (Figure 4). This is also consistent with the fact that the filamentation phenotype of both species in Lee's medium is similar, while it is contrasting in FBS medium. The fraction of species‐specific genes that are differentially expressed was close to 10% in both media. These fractions are slightly smaller than the overall proportion of expressed species‐specific genes between these two species (12.5%).

If we only consider the genes that are differentially expressed in the *bcr1* mutant in both inducing conditions, the fraction of gene targets that are shared between the two species is even smaller (10.3%, Figure 4). In total, *C. dubliniensis* had 727 differentially expressed genes across conditions, with 3.2% being species‐specific. In *C. albicans* , the overlap between conditions was of 434 differentially expressed genes, of which 2.5% were species‐specific. Performing GO enrichment analysis on the sets of genes that changed their expression in both conditions, we observed that the enriched categories are very different between the two species; only one category (*ribonucleoprotein complex subunit organization*, GO: 0071826) out of 36 was enriched in both species (Figure 4). The category *filamentous growth* (GO: 0030447) was only enriched in *C. dubliniensis* and for genes that are downregulated. This suggests that Bcr1 is needed to transcriptionally induce the filamentation program in this species. In *C. albicans* , enriched functions in downregulated genes are instead related to the cell cycle (GO: 0000278 and GO: 0007049) and *microtubules‐based process* (GO: 0007017).

### 2.7 Known Filamentation Genes Are Differentially Expressed When *BCR1* Is Deleted in the Two Species

To narrow down the observed gene expression differences of the *bcr1* mutants to the genes that are important for filamentation, we first employed previous filamentation expression profiles (O'connor et al. 2010). In these datasets, filamentation‐specific genes were defined by comparing gene expression before and after the induction of filamentation with FBS (Experimental Procedures). Considering our expression profiles determined in FBS, of the 1382 genes that were differentially expressed in the *bcr1* mutant of *C. dubliniensis*, 23.8% were filamentation‐specific. On the other side, from the previously defined filamentation‐specific genes, the expression of 69.6% was not significantly affected in the *bcr1 C. dubliniensis* mutant. Including only genes that change their expression more than two‐fold in the *bcr1* mutant, the number of filamentation‐specific genes remained similar (22.6%). In *C. albicans* , filamentation transcription profiles induced by FBS have only been conducted in rich medium and were therefore not considered. Overall, these results showed that Bcr1 controls several other cellular processes apart from filamentation, but also that a considerable fraction of the filamentation program is independent of this TR.

In addition to filamentation‐specific genes defined by their expression, we also curated a list of genes that have been implicated in filamentation based on previous literature and analyzed their expression in the *bcr1* mutants (Table S4). Among the TRs in this set of manually curated genes, we observed that the negative regulator Nrg1 is upregulated in both species and both conditions in the *bcr1* mutant. This is consistent with the notion that Bcr1 regulates this TR as it binds to its promoter region (Guan et al. 2013). The change in expression of *NRG1* is larger in *C. dubliniensis* when induced with FBS (3.18 log2 vs. 1.40 log2), but, conversely, in Lee's medium *C. albicans* exhibits greater upregulation (1.66 log2 vs. 0.74 log2). These findings support the idea that in *C. dubliniensis* additional Nrg1‐independent regulators are involved in repressing hypha formation when nutrients are present (O'connor et al. 2010; Sullivan and Moran 2011). Interestingly, despite the overexpression of *NRG1* in the mutants of both species, the downstream filamentation regulator Ume6 was only downregulated in *C. dubliniensis* and in both inducing conditions. In *C. albicans* the expression of *UME6* is known to be repressed by Nrg1 and differences in its expression have been previously associated with the filamentation dissimilarities between the two species (O'connor et al. 2010). Similarly, Brg1, another well‐known regulator of filamentation in *C. albicans* , was downregulated in the *C. dubliniensis* mutant in both conditions and only slightly in serum for the *C. albicans* mutant. It has been suggested that hyphal development requires the upregulation of *BRG1* and the associated histone deacetylase Hda1, which together remodel the chromatin state of the hyphal‐associated gene promoters and repress Nrg1 (Parvizi Omran et al. 2022; Su et al. 2013). *TEC1* was also downregulated in the *bcr1* mutant of *C. albicans* under both inducing conditions and more strongly in *C. dubliniensis* but not when inducing with Lee's medium. This filamentation TR has been reported to be regulated by Bcr1, but also to feedback regulate *BCR1* expression (Guan et al. 2013).

Apart from *UME6* and in agreement with the enrichment of the GO category *filamentous growth* among the downregulated genes in *C. dubliniensis*, there were several genes previously associated with filamentation that were downregulated in this species in both conditions and that did not show an expression change in *C. albicans* (Table S4). Among the best studied of these genes are *ALS1*, *DEF1* (both very strongly downregulated), *CPH1*, and *RIM101*. Similarly, *HWP1*, encoding a hyphal cell wall protein, was downregulated in the mutant of both species and under both conditions, but much stronger in *C. dubliniensis* (> 9 log2 fold in *C. dubliniensis* vs. < 1.7 log2 fold in *C. albicans* ). In contrast, there were relatively few filamentation‐associated genes that were only upregulated in *C. dubliniensis*, such as *PHO4* and *RBF1*. The gene *RHD3* changed its expression considerably in the mutants of both species, but it did so in different directions.

There were also filamentation‐associated genes that only changed their expression in the *C. albicans* mutant in both inducing conditions, and most were downregulated (*RFX2*, *OPI1*, *ALS3*, *RBT5*, and *ECE1*). The case of *ECE1* is interesting as this gene encodes Candidalysin, a hyphal‐specific toxin that is needed for gut colonization (Liang et al. 2024). Among the most conspicuous genes due to the magnitude of their expression change, even when they only changed in one of the inducing conditions, were *SAP4* and *ALS2* in *C. albicans* and *SFL2* in *C. dubliniensis*, all three only changed in Lee's medium. Overall, in addition to the specific expression differences between the two species in key filamentation regulators and genes such as *UME6*, *BRG1* and *TEC1*, our results revealed large‐scale contrasts in the transcription programs controlled by Bcr1 during filamentation in these two species.

## 3 Discussion

The morphological transition between yeast cells and hyphae is an essential trait for the colonization of the human body by pathogenic fungal species such as *C. albicans* . Dissimilarities in this transition could explain virulence differences between closely related species, as has been suggested for *C. albicans* and *C. dubliniensis* (Moran et al. 2012). The wider range of environmental conditions that have been observed to trigger filamentation in *C. albicans* seems to be associated with its higher clinical prevalence. At a molecular level, TRs such as Nrg1, Ume6, and Efg1 have been identified to underlie part of these differences (Caplice and Moran 2015; Moran et al. 2007; O'connor et al. 2010). However, there are filamentation dissimilarities between these two species that seem to be independent of these TRs. To further understand the molecular underpinnings of the filamentation differences between *C. albicans* and *C. dubliniensis*, we focused on all the TRs that had been previously implicated in filamentation in *C. albicans* , the most studied species. The orthologs of the 45 TRs in the two species are highly similar at the sequence level, containing the same protein domains. However, analysis of a few available DNA binding motifs that these TRs bind revealed extensive differences in their genomic distribution between species. Although it is possible that differences in the coding region and DNA binding affinity of the TRs are important for the filamentation differences between *C. albicans* and *C. dubliniensis*, our observations suggest that at the distance between *C. albicans* and *C. dubliniensis* (∼20 million years) most evolutionary changes occurred in the target genes that they control through *cis*‐regulatory mutations. This agrees with previous observations in the transcription circuits that regulate biofilm formation and glycolysis in these two species (Mancera et al. 2021; Singh‐Babak et al. 2021).

The general high sequence similarity between ortholog TRs and low conservation of the distribution in DNA binding motifs also showed that without experimentation it would be difficult to pinpoint further TRs that are responsible for the filamentation differences between *C. albicans* and *C. dubliniensis*. For this reason, we generated a collection of knockout mutants of the *C. dubliniensis* orthologs of the 45 *C. albicans* filamentation TRs. The only TR for which we could not delete both alleles was Tup1, despite several knockout attempts. This was surprising since there are reported mutants of this TR in *C. albicans* and *C. tropicalis* , the two most closely related species (Braun and Johnson 1997; Gong et al. 2019). In *C. albicans* , Tup1 acts as a repressor of filamentation genes by interacting with the DNA‐binding TR Nrg1, despite not directly binding DNA itself (Braun et al. 2001). One of the explanations for our inability to knockout *TUP1* in *C. dubliniensis* is that this gene has gained a more general role in this species and has thus become essential. Further work will be needed to elucidate the role of *TUP1* in *C. dubliniensis* filamentation and its overall cellular physiology.

Functional characterization of the *C. dubliniensis* gene‐knockouts in parallel with the corresponding *C. albicans* mutants revealed several TRs with contrasting filamentation phenotypes, beyond previously known differences (Figure 2 and Figures S1 and S2). Validating our approach, we observed phenotypic interspecific differences in the *nrg1* and *ume6* mutants, the two TRs that have been previously more strongly associated with the filamentation dissimilarities between these species (Moran et al. 2007; O'connor et al. 2010). On the contrary, we did not observe differences in the *efg1* mutants. This TR has been proposed to regulate several filamentation genes that are only differentially expressed in *C. albicans* (Caplice and Moran 2015). However, the role of Efg1 in biofilm formation is known to be conserved between the two species (Mancera et al. 2021), which would agree with the conservation that we observed in terms of the filamentation defects of the *efg1* mutants in both species (Figure S2).

Among the most studied TRs that, to our knowledge, had not been previously associated with filamentation differences between the two species are *BCR1* and *BRG1*; the mutants of these TRs showed reduced filamentation only in *C. dubliniensis* under the inducing conditions of the screen. In agreement, Bcr1 is known not to be required for filamentation in white *C. albicans* cells, but to be a repressor of this morphological transition in opaque cells (Guan et al. 2013). In contrast, Brg1 is a known regulator of filamentation in the white *C. albicans* state, while it does not seem to be important for hype formation in opaque cells (Guan et al. 2013). Furthermore, both of these TRs are central for biofilm formation in both species (Mancera et al. 2021). Other mutants with marked differences included *rca1*, whose phenotype is similar to that of the reference strain in *C. dubliniensis*, while it was hyperfilamentous in *C. albicans* , resembling the *nrg1* mutant. Deletion of *STP2* was also contrasting, as the mutant did not filament in *C. dubliniensis* while it did in *C. albicans* , although forming unusually thick filaments and cells. Overall, our screen showed that several TRs are responsible for the differences in filamentation between these two species and suggest key TRs for future work.

Bcr1 was originally described as a zinc finger TR needed for biofilm formation in *C. albicans* , but that was dispensable for filamentation (Nobile and Mitchell 2005). This was surprising given that biofilm formation and filamentation are tightly interconnected processes. *BCR1* was later associated with filamentation, although only in opaque cells (Guan et al. 2013). Therefore, we did not expect to observe a filamentation defect in the *bcr1* mutant of *C. dubliniensis*, as we did. Further characterization of the *bcr1* mutants in a variety of inducing media showed that the filamentation defect was condition specific. We found media in which the *C. albicans* mutant did show a filamentation defect, but also in which the *C. dubliniensis* knockout was able to filament (Figure 3, Figure S3). The general observed trend, however, is that there were more inducing conditions in which the *C. albicans* mutant did not show a phenotype when compared to the *C. dubliniensis* mutant. It is important to consider that the deletion of *BCR1* is known to have different consequences among *C. albicans* strains in terms of biofilm formation and filamentation. These differences have been attributed to the diversity of trans‐acting factors that affect the regulatory relationship between Bcr1 and Brg1 (Huang et al. 2019). Within‐species variation in the phenotypes of *bcr1* mutants has also been observed in *C. parapsilosis* (Pannanusorn et al. 2014) and could, therefore, be expected in *C. dubliniensis*.

In *C. albicans* , the role of Bcr1 has been mostly characterized during biofilm formation. This TR is thought to be mainly associated with the adherence of cells to surfaces through the activation of several genes that encode adhesins (e.g., Als1, Als3 and Hwp1) (Lohse et al. 2018; Pannanusorn et al. 2014). These cell‐wall proteins are not only key for the initial establishment of the biofilm but are also expressed throughout latter stages, promoting interactions among hyphae. Thus, although Bcr1 is not essential for hyphal morphogenesis, it is believed to be necessary for the formation and structural integrity of the filamentous biofilm layer (Lohse et al. 2018). In line with its role in adherence, Bcr1 is also thought to be needed for the interaction with bacteria in the biofilm (Lohse et al. 2018). More broadly, deletion of *BCR1* has been shown to alter susceptibility to antimicrobial peptides and impair infection in a murine model (Noble et al. 2010; Pannanusorn et al. 2014). Alongside biofilm formation, these processes have important clinical implications. Our findings suggest that Bcr1 fulfills a divergent physiological role in *C. dubliniensis*, highlighting potential species‐specific regulatory mechanisms. Further investigation in *C. dubliniensis* should elucidate whether the transcriptional programs and functional pathways governed by Bcr1 in *C. albicans* are conserved or unique traits.

Transcriptional profiling in two different inducing conditions showed considerable interspecific differences in the genes that changed their expression in the *bcr1* mutant (Figure 4). This holds true even if we only take into account the genes that showed a larger expression change. In fact, using a 1.5 or 2 log2 fold change cutoff reduced the commonly differentially expressed genes between the two species. There are not many studies where genome‐wide gene expression has been compared between *C. albicans* and *C. dubliniensis*, and most compare profiles done in different laboratories (Caplice and Moran 2015; O'connor et al. 2010). However, the overall degree of conservation from experiments done in a single study is not far from what we observed. For example, between 22% and 29% of differentially expressed genes during biofilm formation in these two species was reported to be conserved (Mancera et al. 2021), while we observed 17% and 20% in the two inducing conditions here tested. These results are also consistent with the low degree of conservation in the genomic distribution of the binding motifs of the analyzed TRs. The proportion of commonly expressed genes between *C. albicans* and *C. dubliniensis* is also significantly lower than that reported for *bcr1* mutants across different *C. albicans* strains (∼50% between any two compared isolates) (Huang et al. 2019). However, at this point it is still difficult to say whether the overlap between the transcription programs controlled by Bcr1 in the two species is lower than expected and could underlie the interspecific filamentation differences observed in the mutants.

The transcription profiles revealed several filamentation genes that are influenced by Bcr1 and that could explain the interspecific filamentation differences in the mutants of this TR. Interestingly, deletion of *BCR1* seems to only affect the expression of *UME6* in *C. dubliniensis*. This gene encodes a TR that is an important activator of the filamentation program, and differences in its expression have been previously proposed to partially underlie the hypha formation dissimilarities between *C. albicans* and *C. dubliniensis* (Moran et al. 2011; Sullivan and Moran 2011). Bcr1 has been shown to directly bind the promoter of *UME6* under biofilm‐forming conditions, while it is not bound in *C. albicans* (Mancera et al. 2021), which is in agreement with the expression differences that we observed between the species. *UME6* is also known to be negatively regulated by the filamentation repressor Nrg1, and considerable emphasis has also been placed on this other regulator to explain filamentation in these species. Our experiments suggest that Bcr1 directly or indirectly represses *NRG1* in both species, as its deletion leads to overexpression of this gene, although more strongly in *C. dubliniensis* when induced with FBS. As for *BCR1*, within‐species variation has been observed in the filamentation defects of *ume6* gen‐knockout mutants in *C. albicans* (Huang et al. 2019); further work in different genetic backgrounds will be needed to determine whether this is also the case in *C. dubliniensis*.

Several other filamentation genes had a similar expression pattern to *UME6*, being down regulated only in the *C. dubliniensis bcr1* mutant. In fact, the functional category *filamentous growth* was only enriched in downregulated genes in the mutant of *C. dubliniensis*, suggesting that in this species Bcr1 plays a more central role regulating filamentation. Still, in *C. albicans* , Bcr1 specifically performed as a positive regulator of key hyphal factors such as *ECE1*.

In summary, our work showed that several TRs, beyond previously known ones, seem responsible for the filamentation differences between *C. albicans* and *C. dubliniensis*. In addition, the role of the TRs is condition dependent, reflecting the complexity of the filamentation programs in these species. We propose that Bcr1 plays a more predominant role in the filamentation of *C. dubliniensis*, controlling the expression of key TRs such as Ume6. Overall, the degree of dissimilarities found between *C. albicans* and *C. dubliniensis* at a molecular level may not be that surprising if we consider that we are comparing organisms that diverged at least as early as humans and gibbons did. The results of our filamentation screen and the collection of gene‐deletion mutants will be valuable resources to further understand the virulence differences between *C. albicans* and *C. dubliniensis* in the near future.

## 4 Experimental Procedures

### 4.1 Identification of Transcription Regulators Associated With Filamentation

TRs associated with filamentation in *C. albicans* were identified from the information available at the Candida Genome Database (CGD) (Skrzypek et al. 2017). A TR, as defined by Homann et al. (2009) (Homann et al. 2009), was considered associated with filamentation if it was part of the Phenotype Term “filamentous growth” including the following phenotypes: “filamentous growth: abnormal”, “filamentous growth: increased”, “filamentous growth: decreased”, “filamentous growth: absent,” “filamentous growth: decreased rate,” and “filamentous growth: delayed”. This is a similar strategy to what has been used by Noble et al. (2010) to identify virulence associated genes. *C. dubliniensis* one‐to‐one orthologs were identified from the orthology assignments at the Candida Gene Order Browser (CGOB) (Maguire et al. 2013).

### 4.2 Estimation of the Amino Acid Sequence Identity Between Transcription Regulator Orthologs and Identification of Protein Domains

*C. albicans* (C_albicans_SC5314_version_A22‐s07‐m01‐r177_default_protein) and *C. dubliniensis* (C_dubliniensis_CD36_version_s01‐m02‐r36_orf_trans_all) TRs amino acid sequences were obtained from CGD to be then pairwise aligned using MUSCLE with default parameters (Edgar 2004). DNA binding domains of each TR were identified with the InterProScan 5.57‐90.0 motif finding algorithm (Jones et al. 2014) and the sequence of the motifs of both species was also aligned using MUSCLE.

### 4.3 Computational Determination of Putative Target Genes

Empirically determined DNA binding motifs of *C. albicans* TRs were obtained from PathoYeastract (Pathogenic Yeast Search for Transcriptional Regulators And Consensus Tracking) (Teixeira et al. 2023). The position‐specific probability matrices of these motifs were used to scan the *C. albicans* (C_albicans_SC5314_version_A22‐s07‐m01‐r109_chromosomes) and *C. dubliniensis* (C_dubliniensis_CD36_version_s01‐m02‐r26_chromosomes) intergenic regions using the “Export Location Set Format: MochiView (with optional Motif scoring)” utility of MochiView v1.46 with standard parameters (Homann and Johnson 2010). The algorithm to identify motifs is explained in detail in the MochiView manual. Putative target genes were defined as those having at least one motif in the 2 kb upstream intergenic region. If there was another ORF closer than 2 kb, the limit of the neighboring ORF was set as the end of the intergenic region. Once target genes were defined for each species, targets were compared between species through CGOB orthology assignments.

### 4.4 Generation of the Knockout Mutant Collection of Filamentation Transcription Regulators in *C. dubliniensis*

Homozygous null mutants of the *C. dubliniensis* TRs were generated following the genetic modification strategy previously described by Mancera et al. (2019) (Mancera et al. 2019). In brief, fusion PCR was performed to generate *HIS1* and *LEU2* gene disruption cassettes. To this end, *HIS1* and *LEU2* nutritional markers were amplified from the plasmids pEM001 and pEM002, respectively, with primers 2 and 5. In parallel, approximately 350 nucleotides of the flanking downstream and upstream sequences of the ORF to be deleted were amplified from genomic DNA of *C. dubliniensis* strain CD36 with primers 1–3 and 4–6 in separate reactions. The nutritional markers were then stitched together with the up and downstream homology regions by fusion PCR with primers 1 and 6. To delete the first allele and generate the heterozygous mutant, the *HIS1* disruption cassette was transformed in parallel into CEM074 and CEM075 auxotrophic strains by electroporation (Mancera et al. 2019). The homozygous deletion strain of each TR gene was then generated by transformation of the heterozygous strain with the *LEU2* disruption cassette. Selection of the transformants was performed by growing cells on synthetic defined (SD) medium (6.7 g/L yeast nitrogen base, 2% glucose, supplemented with amino acids) without histidine or leucine. Verification of the correct integration of the deletion cassettes was performed by colony PCR of the 5′and 3′ junctions. In addition, after deleting the second allele, the absence of the target gene was verified with primers that amplify a region within the targeted ORF. Two knockout strains were constructed for each *C. dubliniensis* gene, one in each of the two parental strains CEM074 and CEM075 (Mancera et al. 2019). Strains used in this study are listed in Table S5.

### 4.5 Screening the *C. albicans* and *C. dubliniensis* Mutant Collections for Filamentation Defects

*C. albicans* and *C. dubliniensis* strains were streaked out from glycerol stocks in SD medium without histidine and leucine. A single colony from these plates was used to start liquid cultures in Lee's medium adjusted to pH 4.5 (Lee et al. 1975; O'connor et al. 2010). These cultures were grown at 30°C for 18 h. The cells were then washed twice with Milli‐Q water. Filamentation was induced by inoculating 2 × 10^6^ cells to Milli‐Q H_2_O supplemented with 10% (v/v) FBS (Caplice and Moran 2015; O'connor et al. 2010) on 24‐well cell culture plates. Cultures were incubated at 37°C shaking at 200 rpm. The percentage of filamentous cells was counted under an optical microscope at the time of transferring to inducing condition, and 1 and 3 h after. Three replicate assays were performed for each of the two *C. dubliniensis* mutants of each TR. A Bonferroni multiple corrected *t*‐test was used to compare the percent of filament formation in each TR mutant with that of the reference strain in each species at each time point. At least 10 different view fields were counted per strain, containing a minimum of eight cells per field. For each filamentation TR, two independent mutant strains were characterized for *C. dubliniensis* and one for *C. albicans* . Strains CEM091 and CEM092 were used as a wildtype reference for *C. dubliniensis* (Mancera et al. 2019), and SN250 for *C. albicans* (Noble et al. 2010).

To estimate the MI of the mutants at the 1‐h time point, the length (*l*), maximum diameter (*d*) and diameter at septal junction (*s*) of three representative cells were manually measured using the measurement tool in ImageJ. The index was then calculated with the expression MI = 2 + 1.78 log10 *ls*/*d* ^*2*^ as previously described (Merson‐Davies and Odds 1989). A Bonferroni multiple corrected *t*‐test was used to compare the MI of each TR mutant with that of the reference strain of each species.

To characterize filamentation of the *bcr1* mutants of both species in a wider set of conditions, cultures were grown in Lee's medium at pH 4.5 or pH 7.2, incubating at 30°C for 18 h with constant agitation (Caplice and Moran 2015; O'connor et al. 2010). Filamentation was then induced by inoculating 2 × 10^6^ cells in 24‐well cell culture plates and incubating at 37°C with shaking at 200 rpm. The following nutrient‐rich media were used to evaluate filamentation capacity: YPD and RPMI 1640, both supplemented with 10% FBS (v/v) (O'connor et al. 2010; Vilela et al. 2002), and SD medium with 0.75% glucose, supplemented with 50% FBS. The latter medium has been reported to be optimal to induce filamentation in *C. tropicalis* (Lackey et al. 2013). Low‐nutrient filamentation inducing media employed were Milli‐Q water supplemented with 10% FBS or Lee's medium adjusted to pH 7.2 (Caplice and Moran 2015).

### 4.6 Transcriptional Profiling

For RNA extraction, strains were grown in Lee's medium pH 4.5 at 30°C for 18 h. Cells were then washed twice in Milli‐Q water, and approximately 2 × 10^6^ cells were inoculated in the inducing medium and incubated at 37°C for 1 h, shaking at 200 rpm (Caplice and Moran 2015). The inducing media employed were Milli‐Q H_2_O supplemented with 10% (v/v) FBS or 10% (v/v) Lee's glucose medium pH 7.2, as for the filamentation assays described above. Total RNA was extracted using the RiboPure‐Yeast Kit (Ambion) with some minor modifications. Cells were collected by centrifuging cultures at 3500 rpm for 5 min; the lysis components (Lysis Buffer, 10% SDS and Phenol:Chloroform:IAA) were immediately added to the pellet, and they were then frozen at −80°C. The frozen pellets were thawed on ice, and the rest of the protocol was performed as indicated by the manufacturer, but performing all steps at 4°C in a cold room. Three replicates were performed for each strain (wildtype and *bcr1* mutant) in each inducing medium for each species. Quality control of the total RNA, library preparation, and sequencing were performed by Novogene Co. Libraries were directional using poly A enrichment, and sequencing was PE150 in a NovaSeq platform. A minimum of 2 G raw data per sample were obtained.

To identify differentially expressed genes, raw reads were first cleaned with fastp v0.46.2 (Chen et al. 2018) to remove read regions of low quality, potential adaptor sequences, poly(A)‐tails and long terminal homopolymeric stretches. Clean reads were then aligned and quantified using kallisto v0.46.2 (Bray et al. 2016) against the cDNA transcripts reported in CGD. Strains SC5214 (A22‐s07‐m01‐r168_default_coding) and CD36 (s01‐m02‐r36_orf_coding) were used as references for *C. albicans* and *C. dubliniensis*, respectively. To identify anomalous samples, an outlier map was made based on the robust score distances and orthogonal distances computed by the PCAGrid function (Todorov and Filzmoser 2009) for the normalized count matrix of each dataset, as suggested in (Chen et al. 2020) (Figure S4). Based on these results, two replicates of *C. dubliniensis* in Lee's medium (one for the wild‐type strain and one for the *bcr1* mutant) and three replicates of *C. albicans* (one of the *bcr1* mutant in FBS medium, one of the wild‐type strain in Lee's medium, and one of the *bcr1* mutant in Lee's medium) were removed. A cutoff of 97.5% of the corresponding distribution was used to classify the samples. Finally, the exactTest function of the edgeR package (Robinson et al. 2010) was used to determine gene differential expression. The resulting *p*‐values were corrected with the *q*‐value function using the default parameters to obtain an FDR of 1%. Filamentation‐specific genes based on gene expression were obtained from Table S3 of (O'connor et al. 2010).

The list of differentially expressed genes for each species was then used as input for a GO enrichment analysis using the R package topGO (Alexa 2024). GO terms were mapped to genes using the readMappings function and gene‐to‐GOs association data provided by CGD. For *C. albicans* , the association between genes and GO terms was directly based on *C. albicans* GO annotations. For *C. dubliniensis*, the GO terms were assigned based on the GO annotations of their *C. albicans* orthologs. Enrichment analysis was performed with Fisher's exact test, using the classic algorithm, and a significance cutoff of *p* < 0.01. To summarize the enriched GO terms, we utilized the REVIGO online analysis tool (Supek et al. 2011), allowing a similarity score of 0.7 between GO terms. The UniProt GO database was used for mapping, and SimRel was selected as the semantic similarity measure.

## Author Contributions

**Teresa Meza‐Davalos:** methodology, investigation, visualization, writing – original draft, formal analysis. **Luis F. García‐Ortega:** methodology, investigation, visualization, writing – review and editing, formal analysis. **Eugenio Mancera:** conceptualization, investigation, visualization, funding acquisition, supervision, writing – original draft, formal analysis.

## Disclosure

This manuscript was released as a pre‐print at *bioRxiv*.

## Conflicts of Interest

The authors declare no conflicts of interest.

## Supporting information


**Figure S1.** Micrographs of the mutants for which filamentation could be quantified. Images taken under an optical microscope at the time the cells were transferred to the filamentation inducing conditions (T0) and after one (T1), three (T3), and 5 h (T5) of filamentation. Only one of the *C. dubliniensis* isolates is shown although the phenotype was similar in the other isolate. The reference scale bars represent 10 μm.

**Figure S2.** Phenotypes of the mutants for which filamentation could not be quantified. Micrographs taken under an optical microscope at the time the cells were transferred to the filamentation inducing conditions (T0) and after one (T1), three (T3), and 5 h (T5) of filamentation. Only one of the *C. dubliniensis* isolates is shown although the phenotype was similar in the other isolate. The reference scale bars represent 10 μm.

**Figure S3.** Morphology of the *bcr1* mutant under different filamentation inducing conditions. Micrographs under a light microscope of the reference strain and the *bcr1* mutant of *C. albicans* and *C. dubliniensis* in the different media tested. As indicated at the furthest left, two preculture conditions were employed. Panel A, B, and C show micrographs taken at the 0, 1, and 3 h time points, respectively. The reference scale bar in the wildtype *C. albicans* in H20 + 10% FBS when preculture in Lee^’^s pH 4.5 was used represents 10 μm.

**Figure S4.** Outlier map of the RNA‐seq replicates for the *C. albicans* (CEM093, WT strain and TF137, *bcr1* mutant) in Lee^’^s (a) and FBS (b) media, as well as for *C. dubliniensis* (CEM092, WT strain and CEM106, *bcr1* mutant) in Lee^’^s (c) and FBS (d) media. The black lines represent the cut‐off thresholds for orthogonal distances and the distance score (> 0.975). Replicates above any of the cut‐off thresholds were classified as outliers (indicated by a black arrow) and were excluded from subsequent analyses.

**Table S1.** *C. albicans* filamentation TRs considered in this study.

**Table S2.** Quantitative filamentation characteristics of the mutants. From left to right, the first six columns provide the percentage of the cells that showed filamentous morphologies as in Figure 2. NA represent the mutants that could not be quantified. The last two columns provide the MI of the mutants. Asterix denote the mutants that are statistically different from the wild type (Bonferroni corrected *t*‐test, *p* < 0.05).

**Table S3.** Differential expression results of all ORFs in the *C. albicans* and *C. dubliniensis bcr1* mutant compared to the corresponding wildtype strain. There are four tabs, two correspond to *C. albicans* (Calb) and two to *C. dubliniensis* (Cdub), and for each species there are experiments performed in FBS (FBS) and Lee^’^s (Lees) media. The log2 fold‐change (LogFC), the log2 counts per million (LogCPM), the *p*‐value and the false discovery rate (FDR) are shown for each ORF.

**Table S4.** Expression of genes related to filamentation in the *bcr1* mutants of *C. albicans* and *C. dubliniensis*. Values of genes whose differential expression was not statistically significant according to the FDR, irrespectively of the log2 fold change (FC), are not provided.

**Table S5.** Yeast strains used and generated in this study.

## Data Availability

The raw RNAseq data have been deposited at the NCBI under BioProject ID PRJNA1163420.
