# Supplementary material for: Filamentation Profiling Reveals Multiple Transcription Regulators Contributing to the Differences Between Candida albicans and Candida dubliniensis
Source: Mol Microbiol. 2025 Jul 17;124(4):327–41. doi: 10.1111/mmi.70012 (PMC12510621; doi:10.1111/mmi.70012)
Supplement: Supplementary file 1 — Figure S1. Micrographs of the mutants for which filamentation could be quantified. Images taken under an optical microscope at the time the cells were transferred to the filamentation inducing conditions (T0) and after one (T1), three (T3), and 5 h (T5) of filamentation. Only one of the C. dubliniensis isolates is shown although the phenotype was similar in the other isolate. The reference scale bars represent 10 μm. [file MMI-124-327-s006.pdf]

Supplementary Figure 1

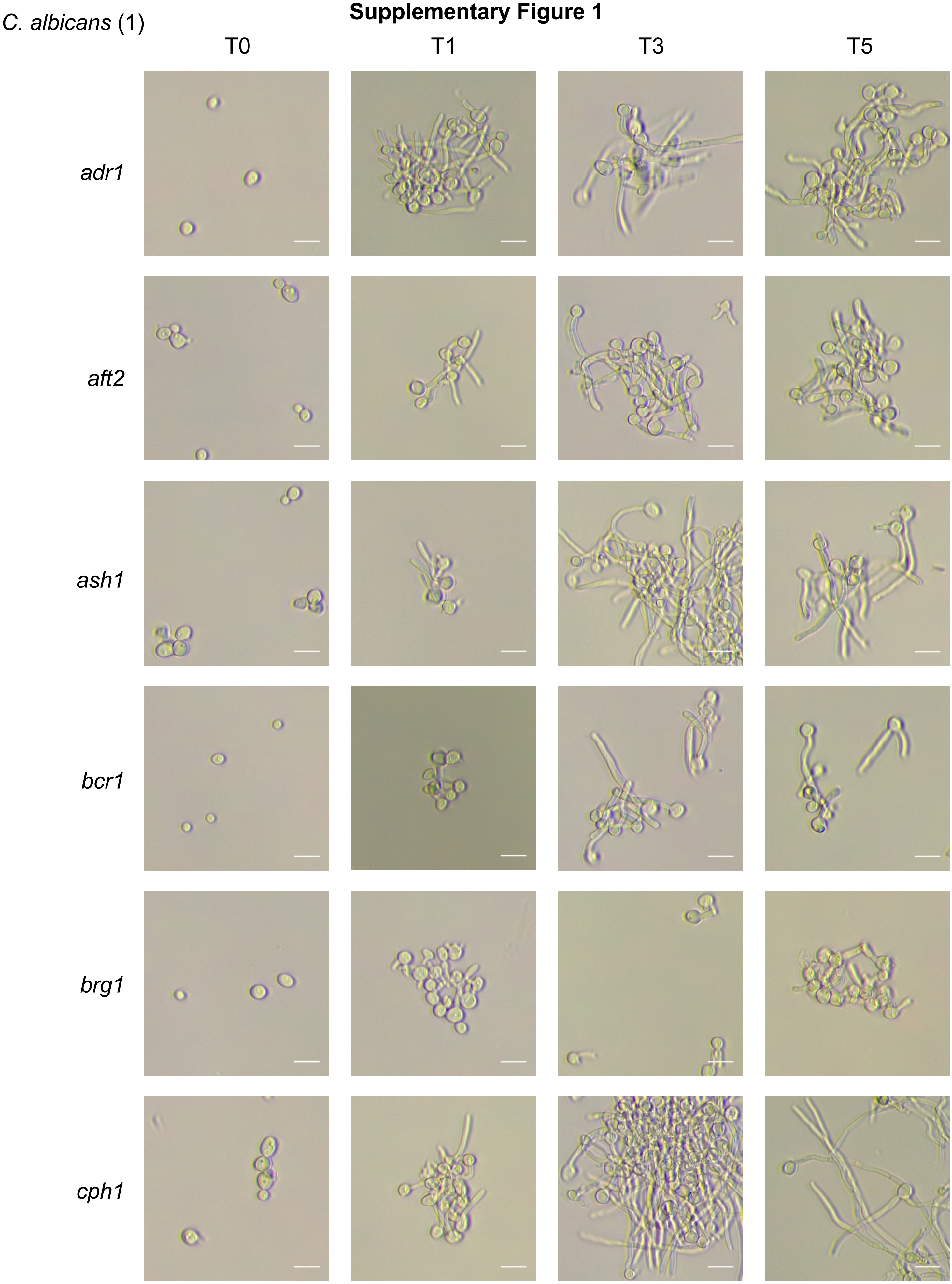

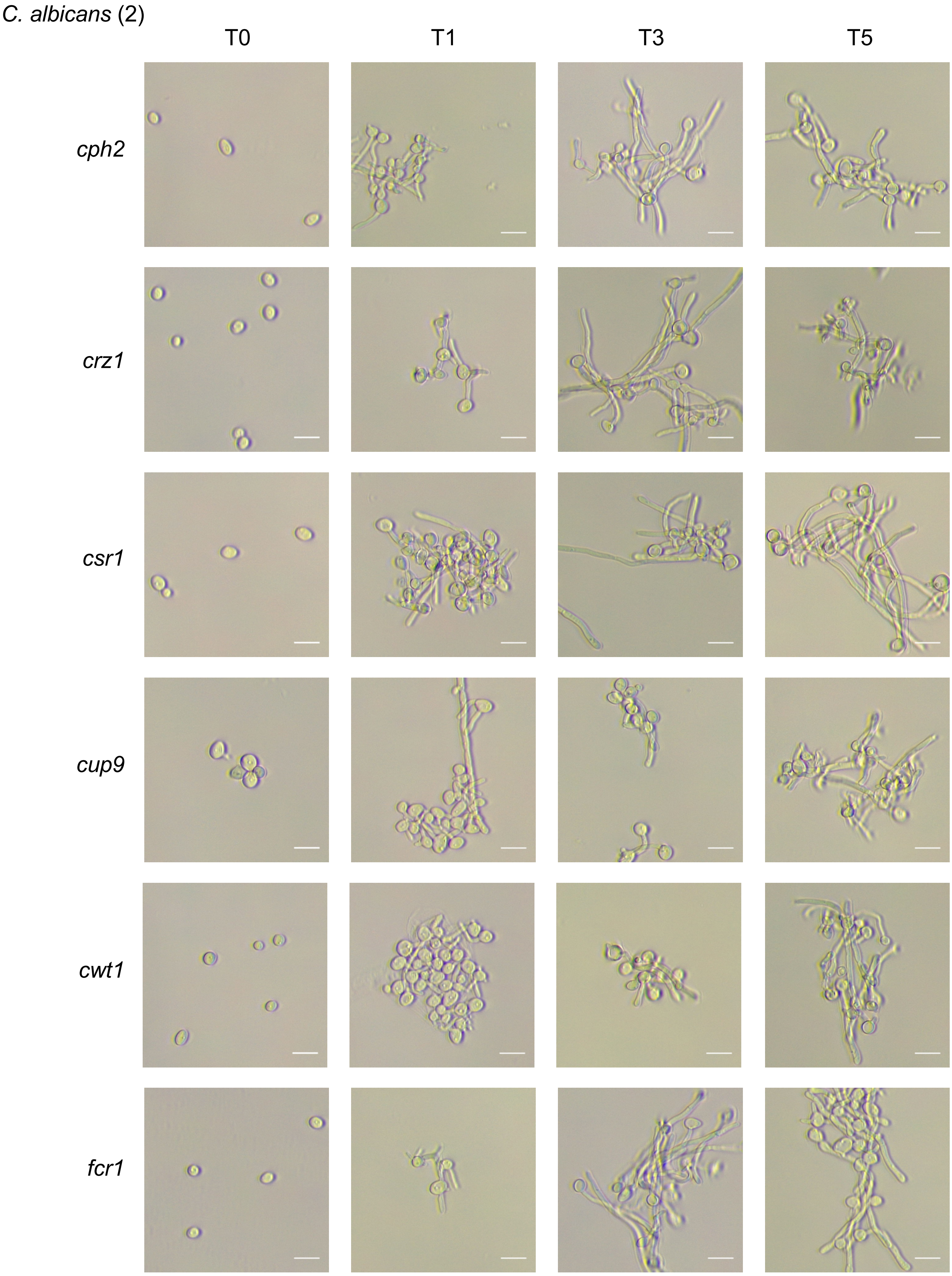
C. albicans (2)
T0
T1
T3
T5
cph2
crz1
csr1
cup9
cwt1
fcr1

C. albicans (3)

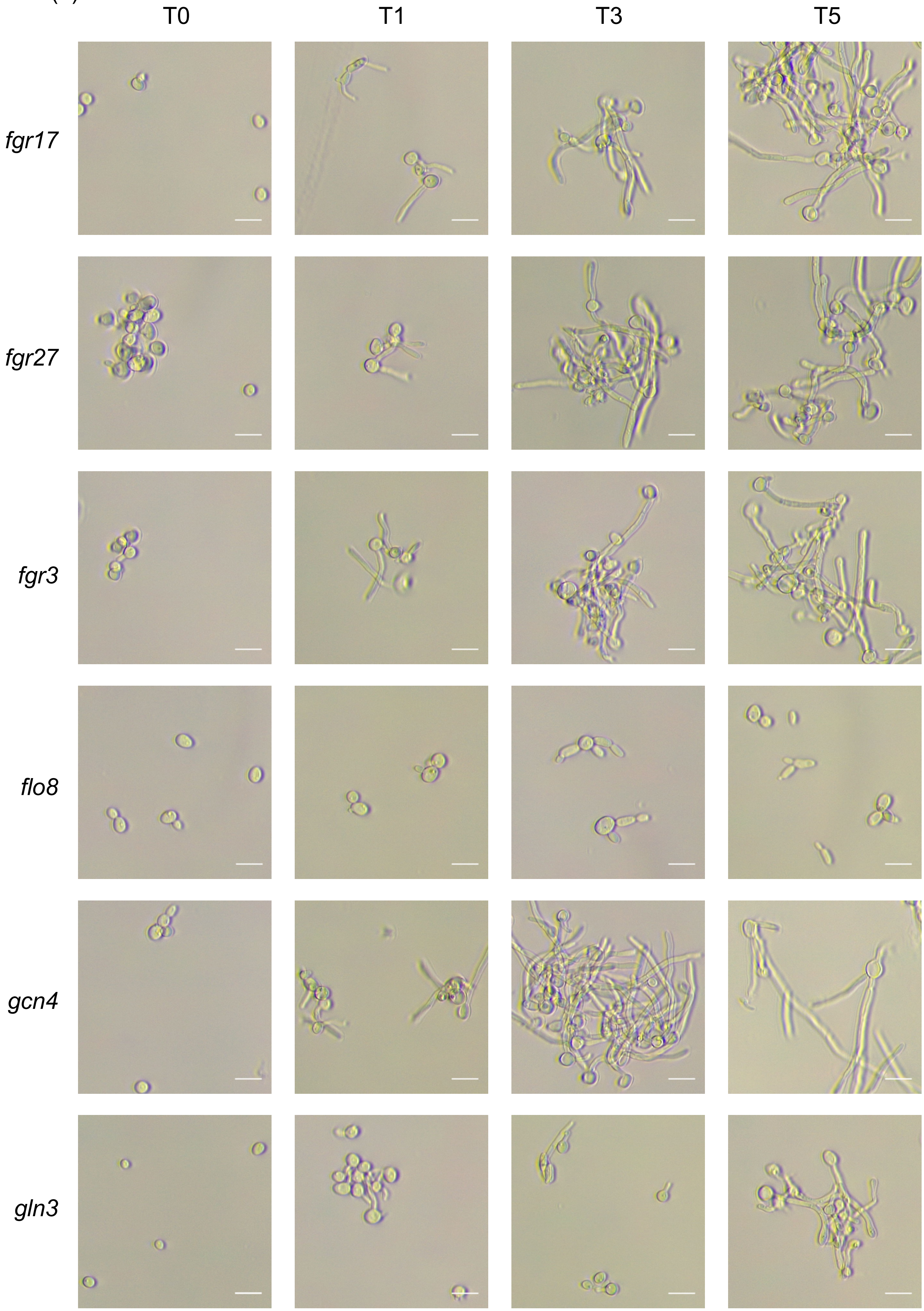

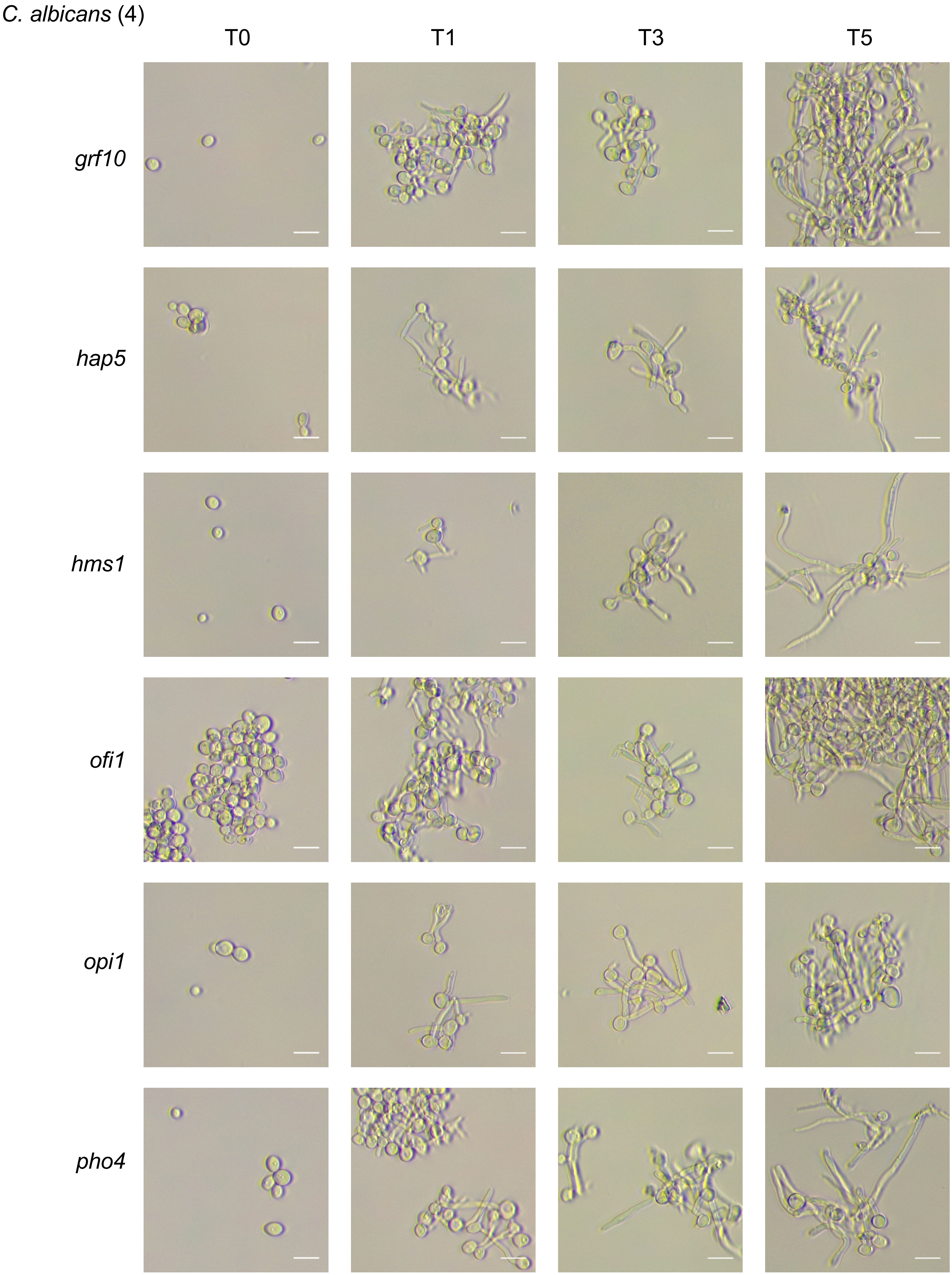
C. albicans (4)
T0
T1
T3
T5
grf10
hap5
hms1
ofi1
opi1
pho4

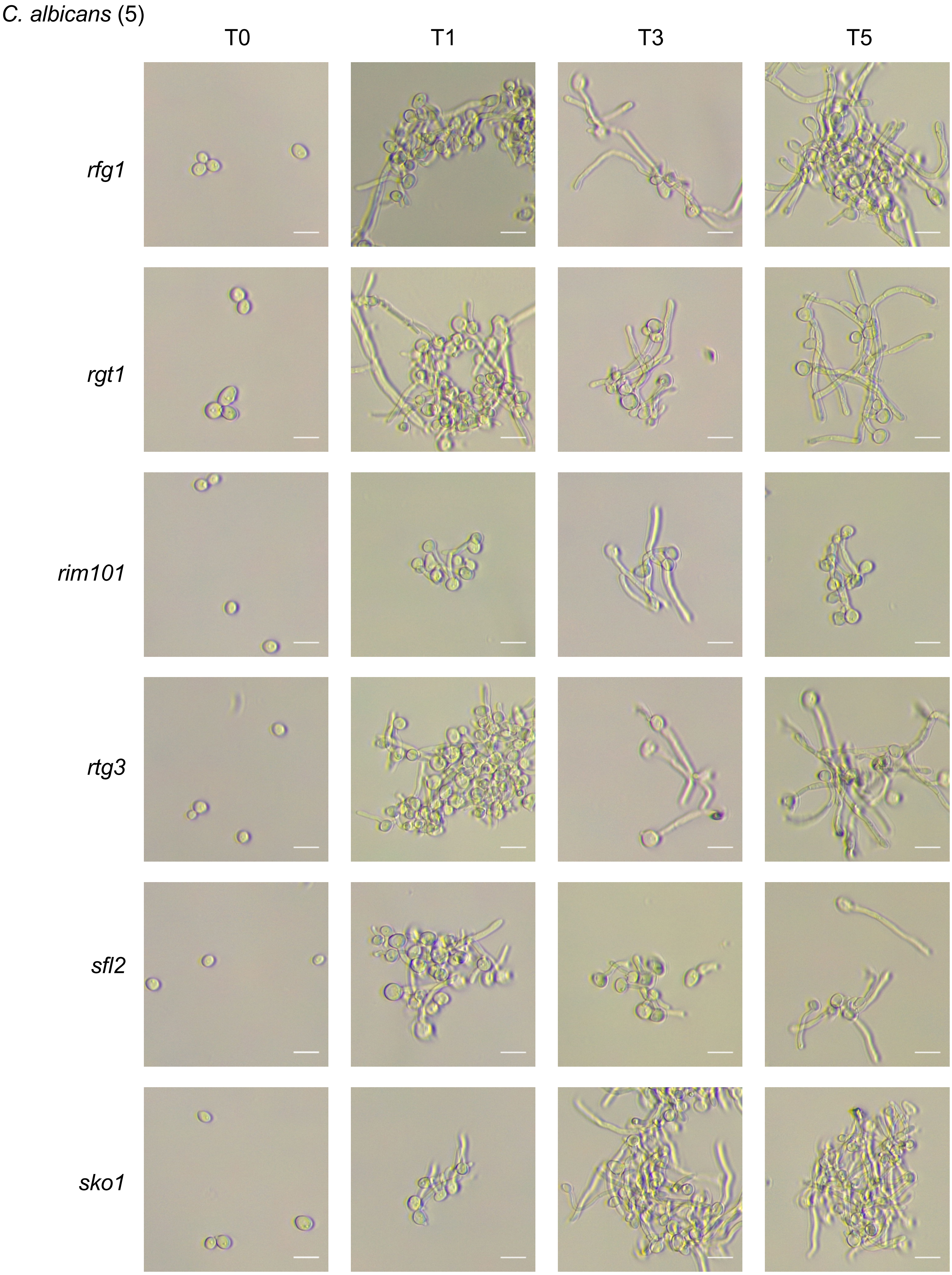
C. albicans (5)
T0
T1
T3
T5
rfg1
rgt1
rim101
rtg3
sfl2
sko1

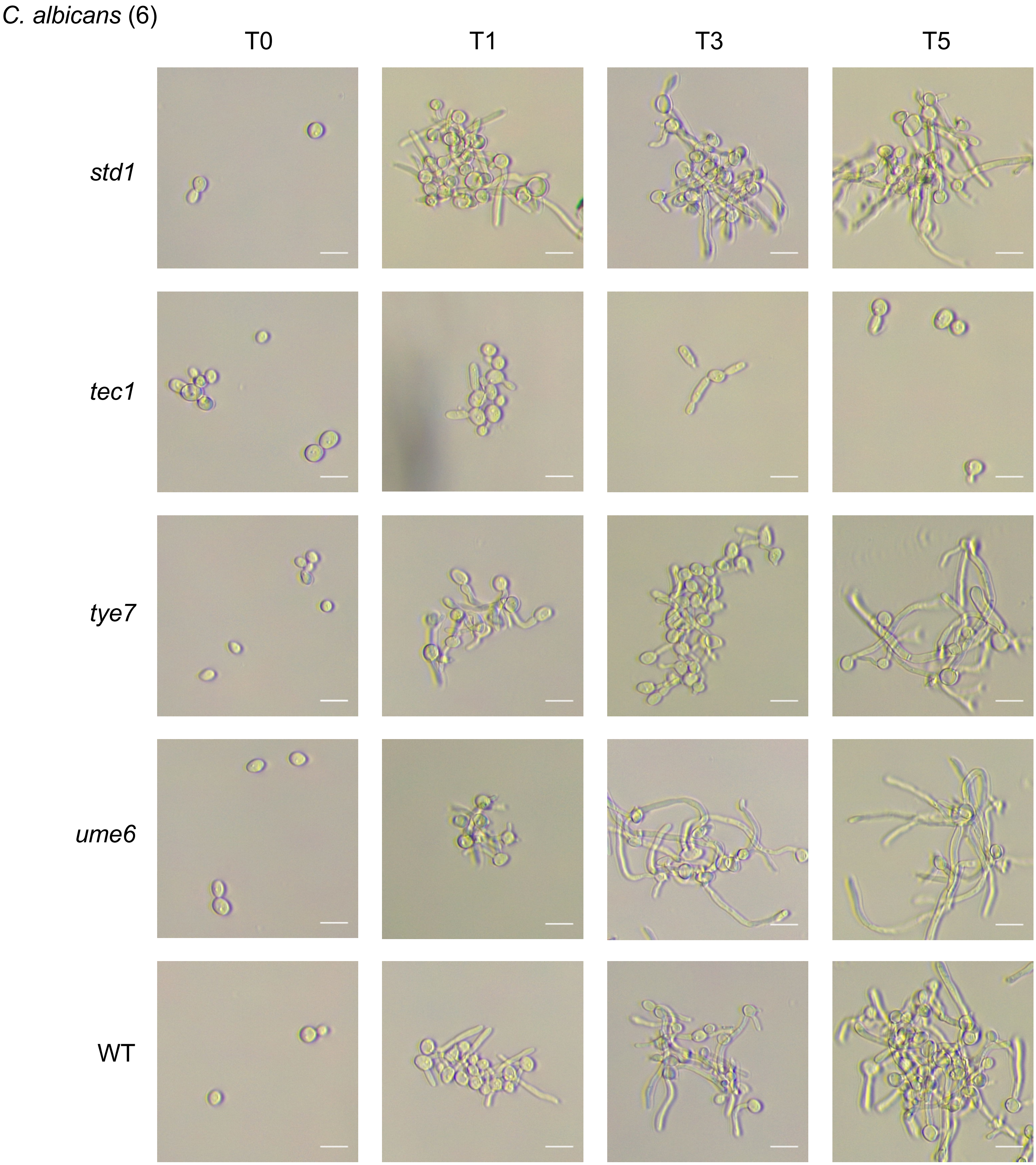

C. albicans (6)
T0
T1
T3
T5
std1
tec1
tye7
ume6
WT

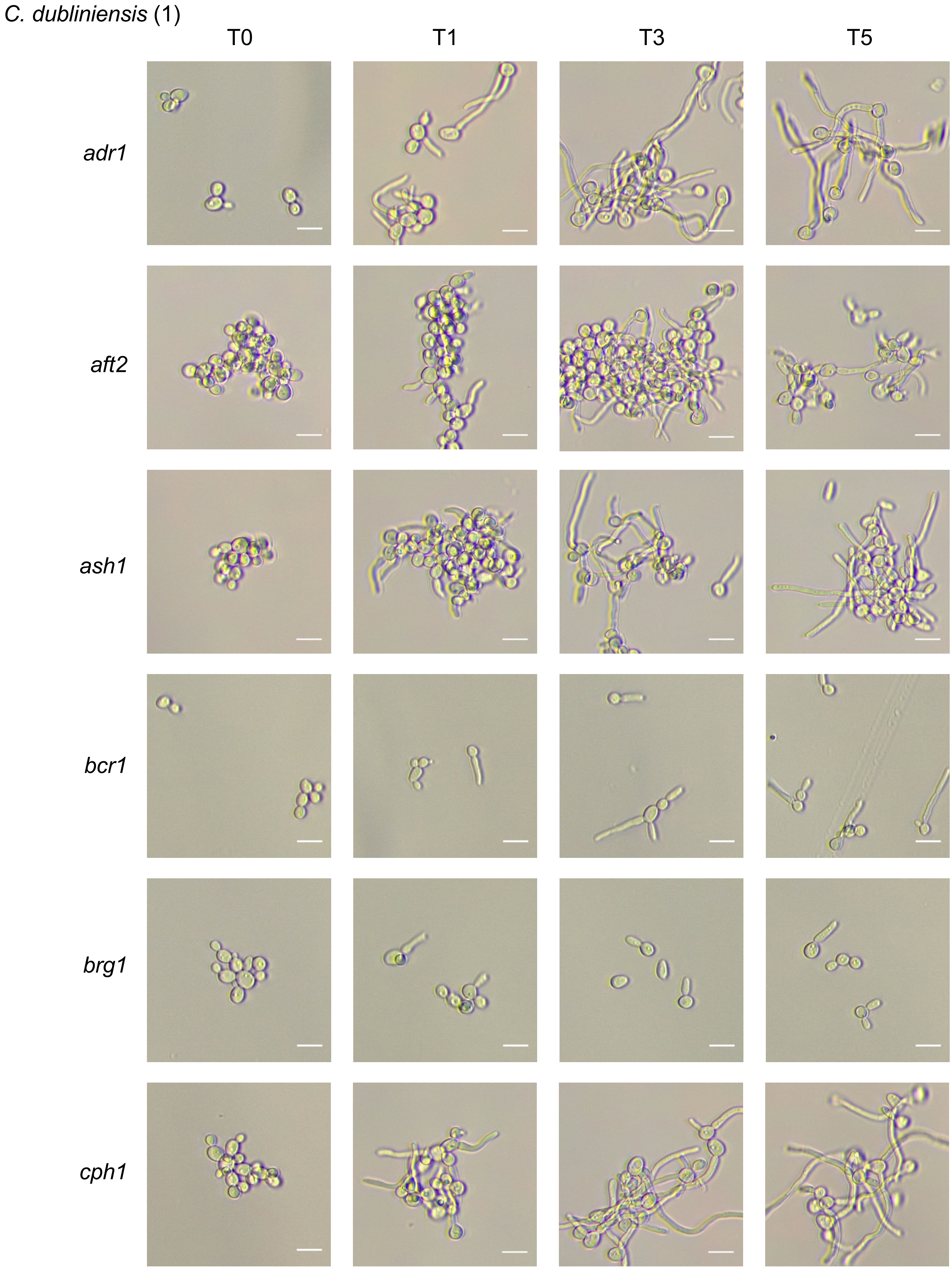
C. dubliniensis (1)
T0
T1
T3
T5
adr1
aft2
ash1
bcr1
brg1
cph1

C. dubliniensis (2)

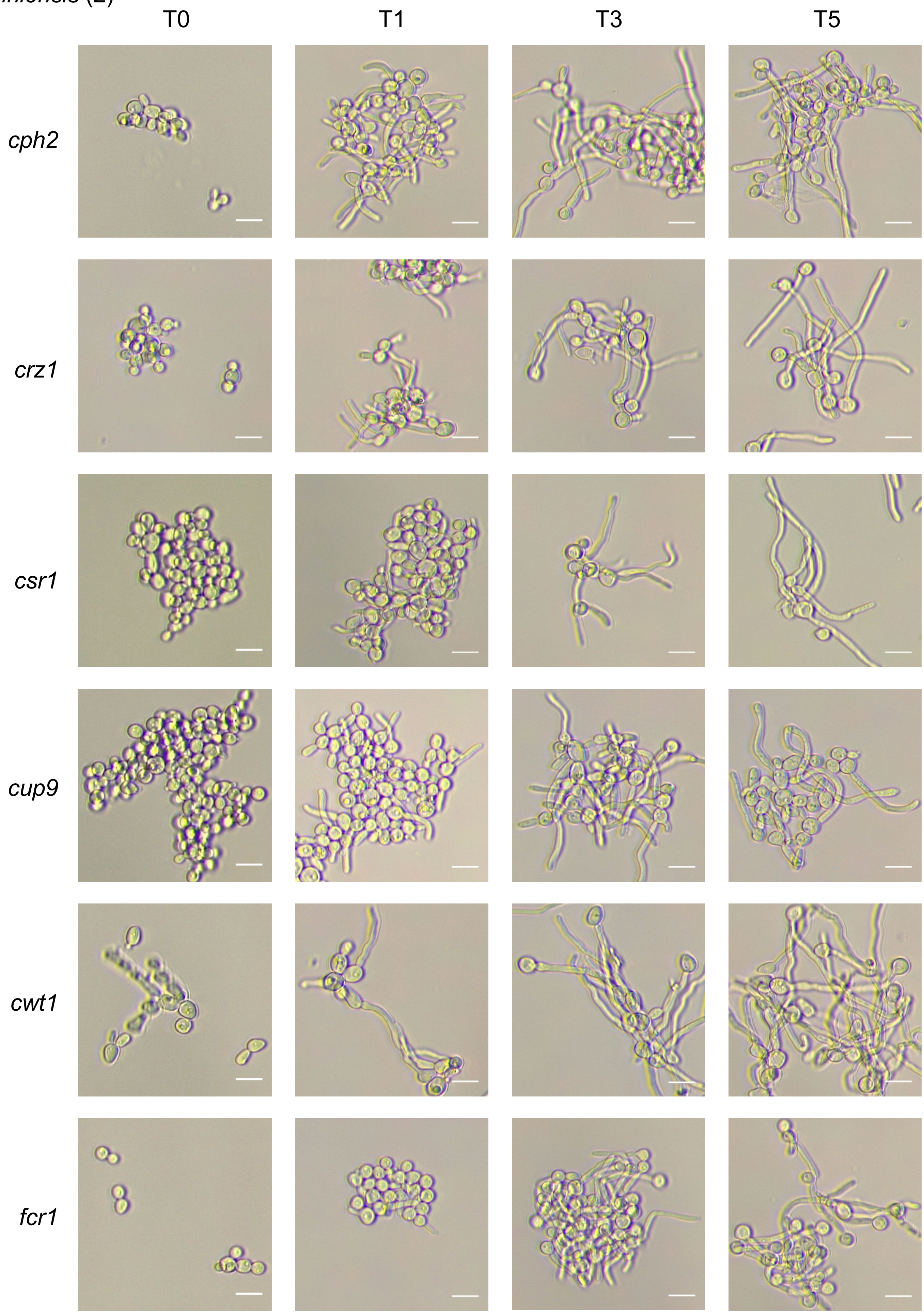

C. dubliniensis (3)

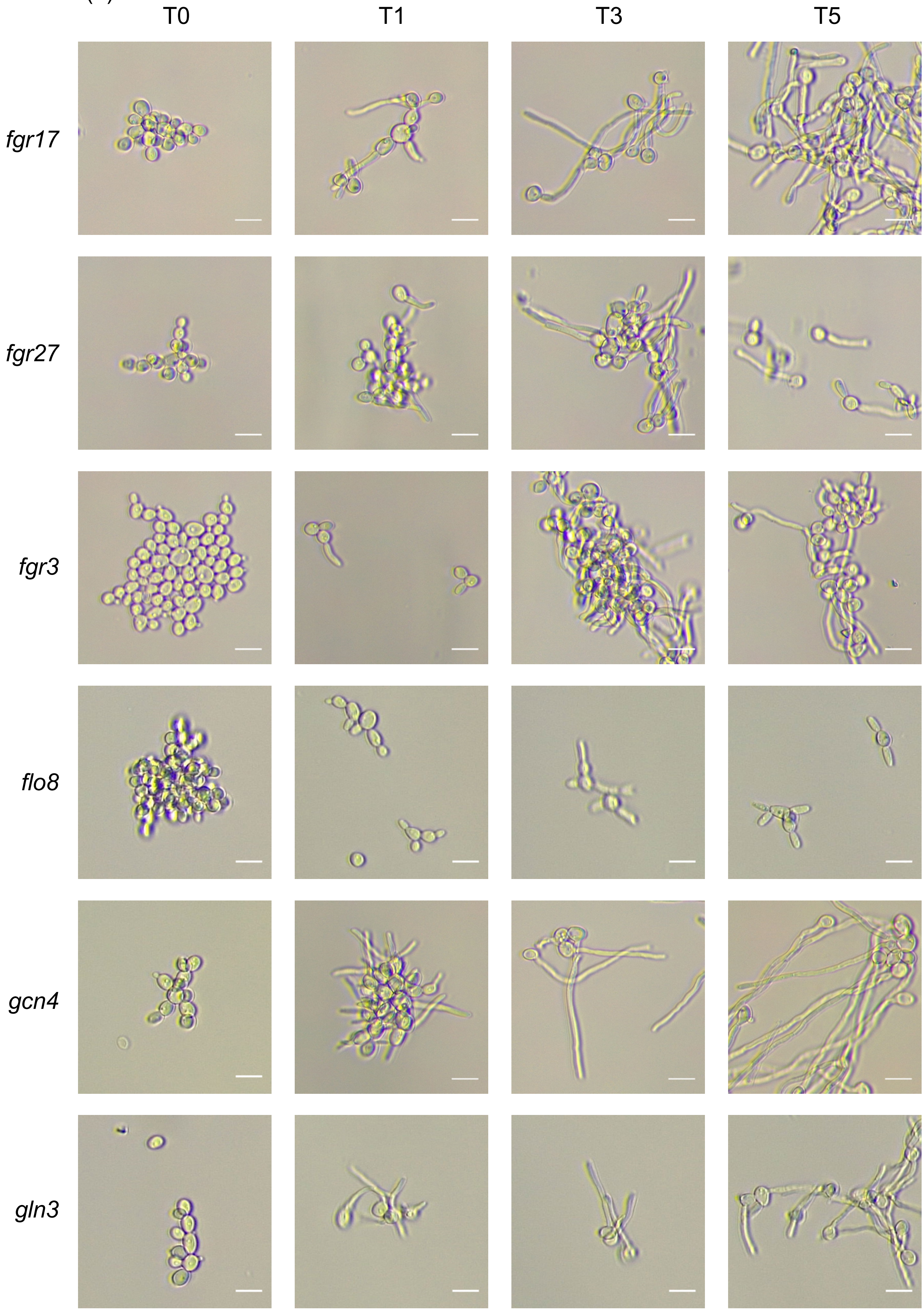

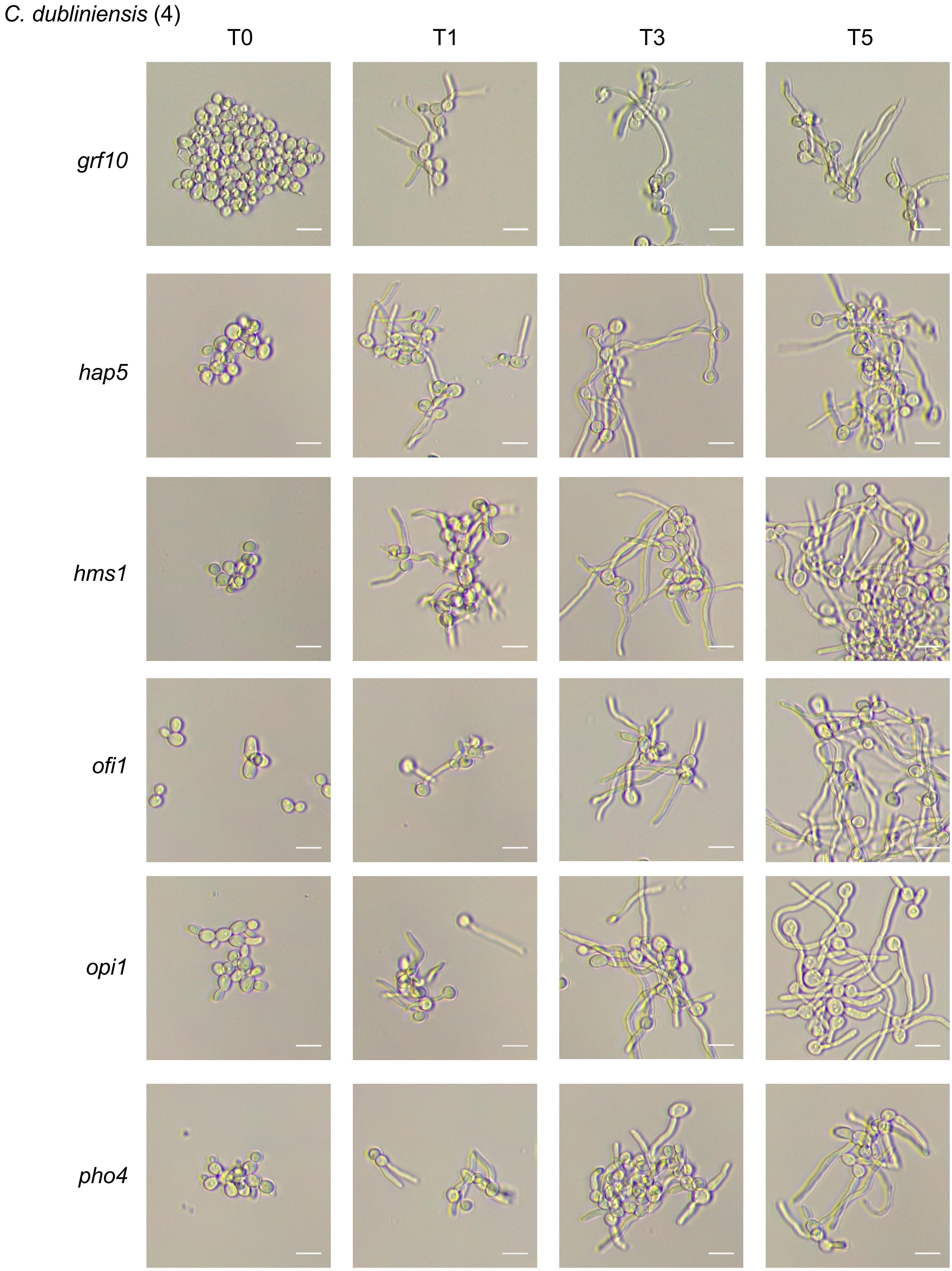
C. dubliniensis (4)
T0
T1
T3
T5
grf10
hap5
hms1
ofi1
opi1
pho4

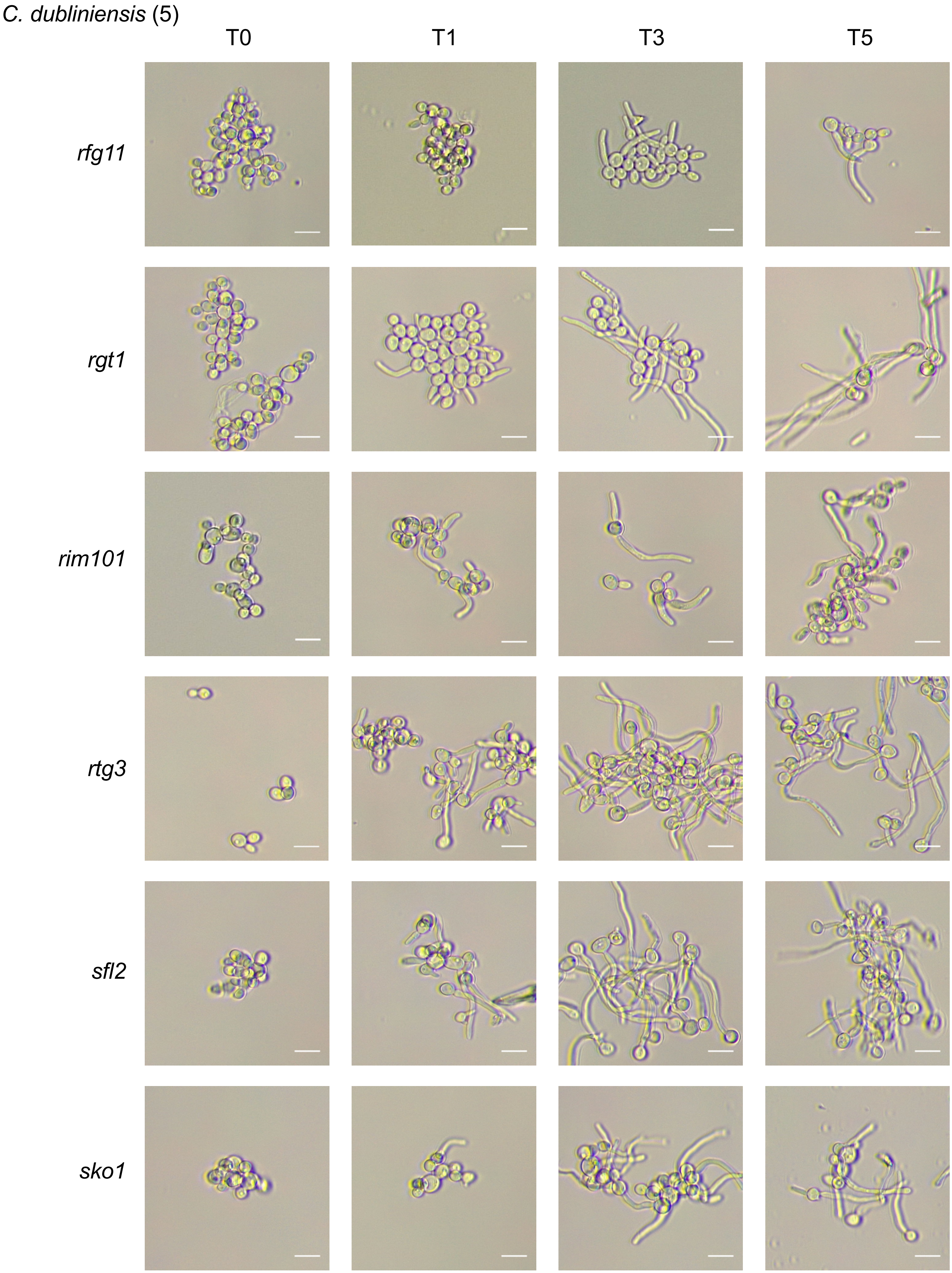
C. dubliniensis (5)
T0
T1
T3
T5
rfg11
rgt1
rim101
rtg3
sfl2
sko1

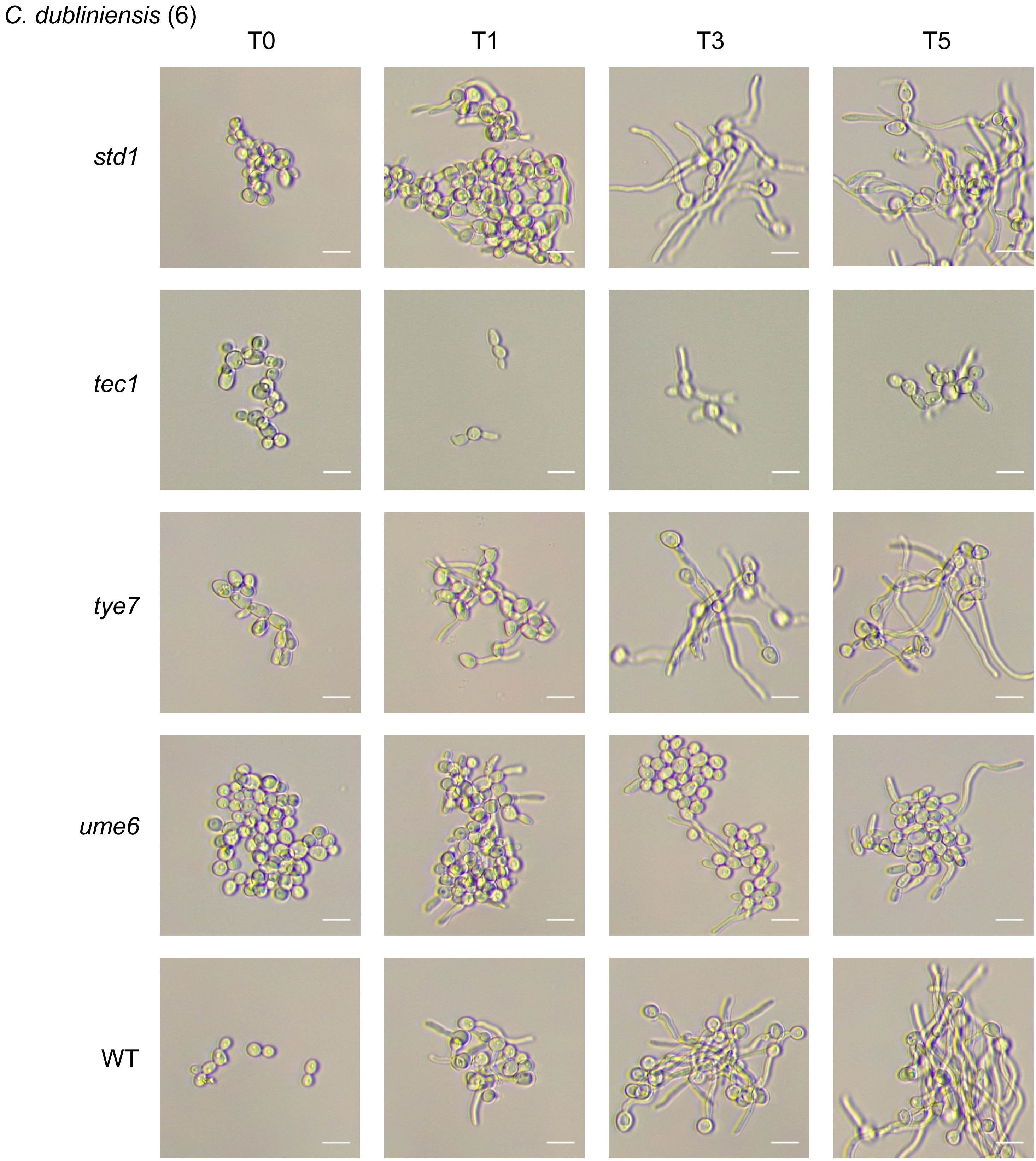

C. dubliniensis (6)
T0
T1
T3
T5
std1
tec1
tye7
ume6
WT
