# Supplementary figures and images for: Filamentation Profiling Reveals Multiple Transcription Regulators Contributing to the Differences Between Candida albicans and Candida dubliniensis
Source: Mol Microbiol. 2025 Jul 17;124(4):327–41. doi: 10.1111/mmi.70012 (PMC12510621; doi:10.1111/mmi.70012)

*C. albicans* (1)

Supplementary Figure 2

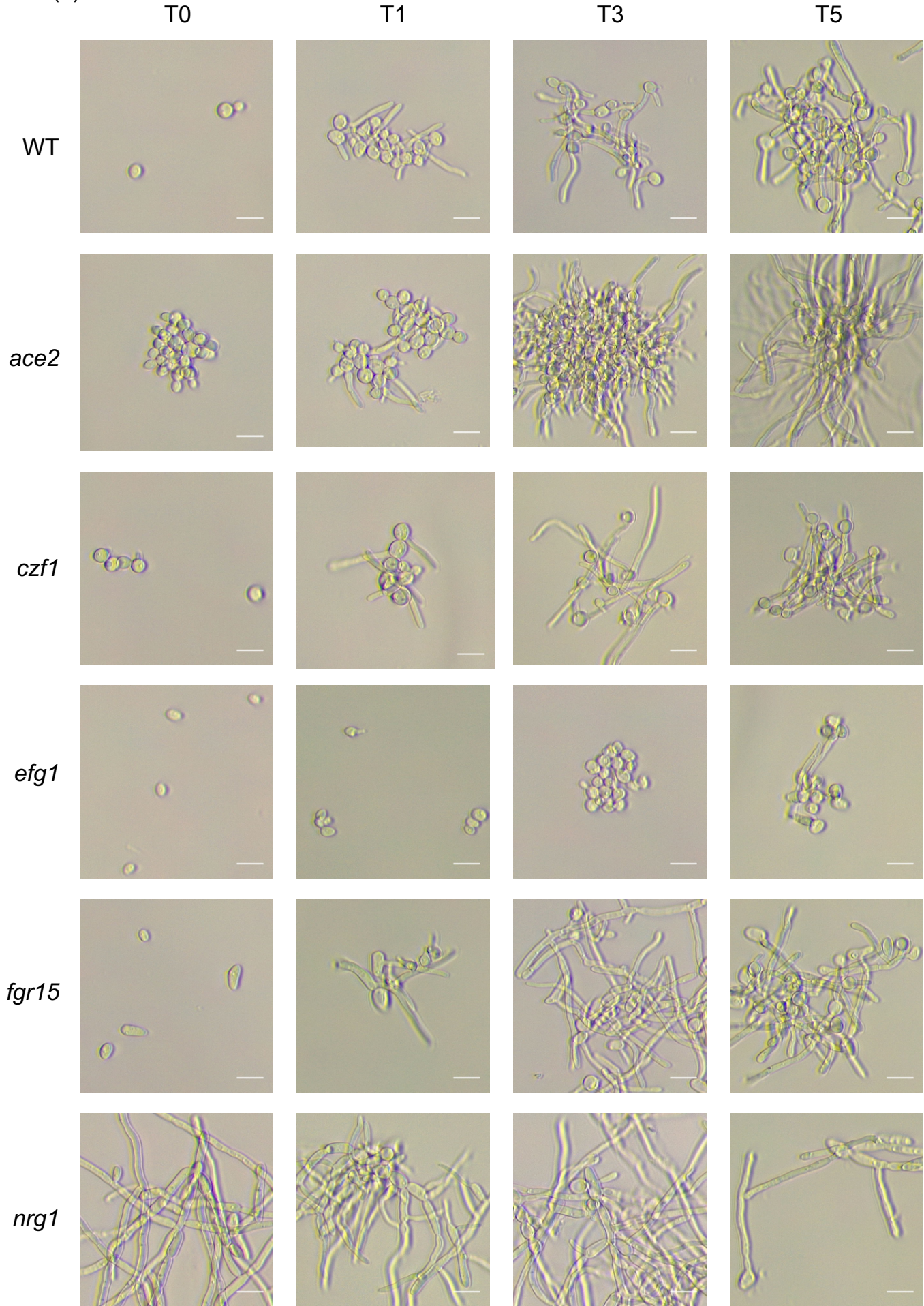

*C. albicans* (2)

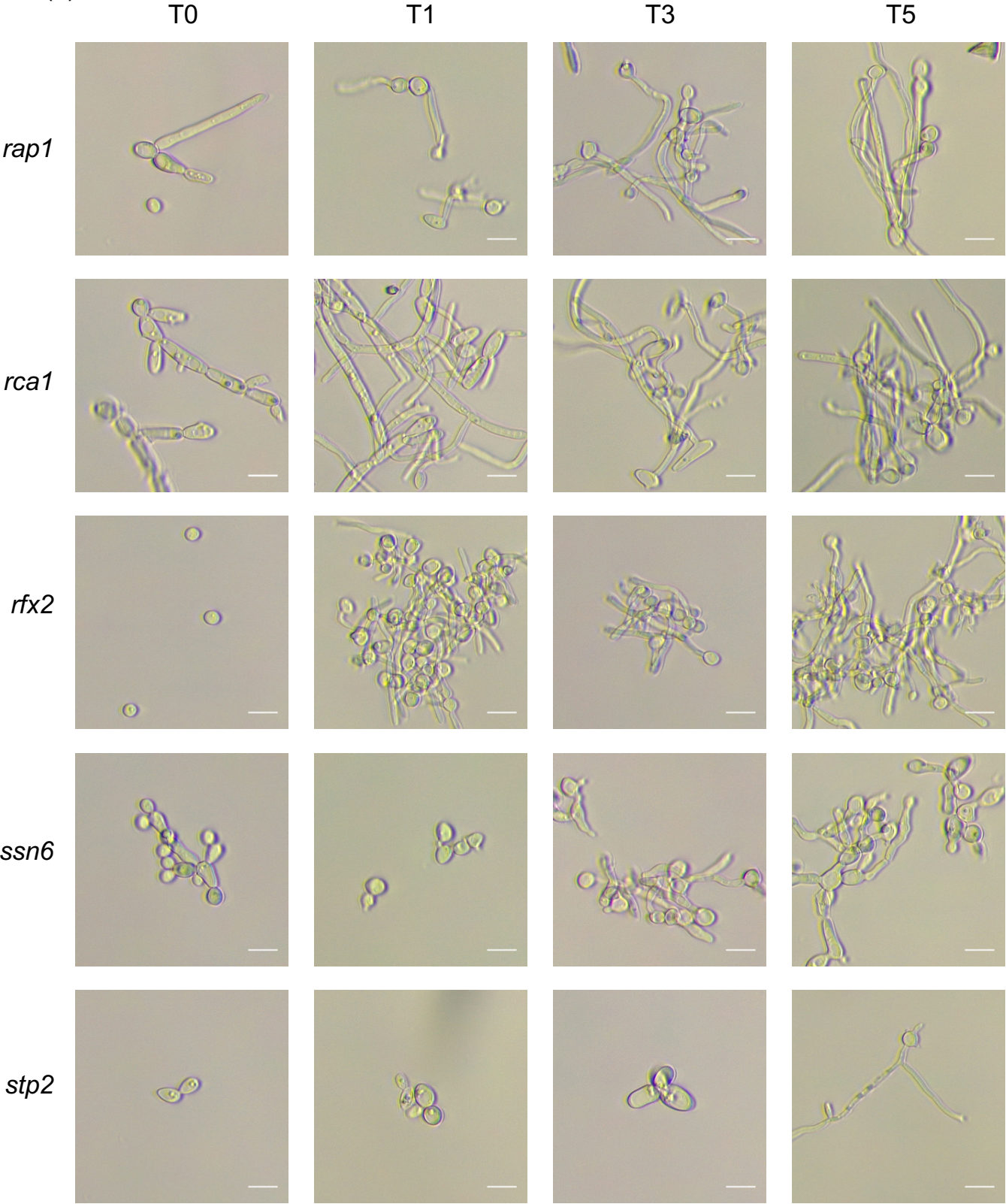

*C. dubliniensis* (1)

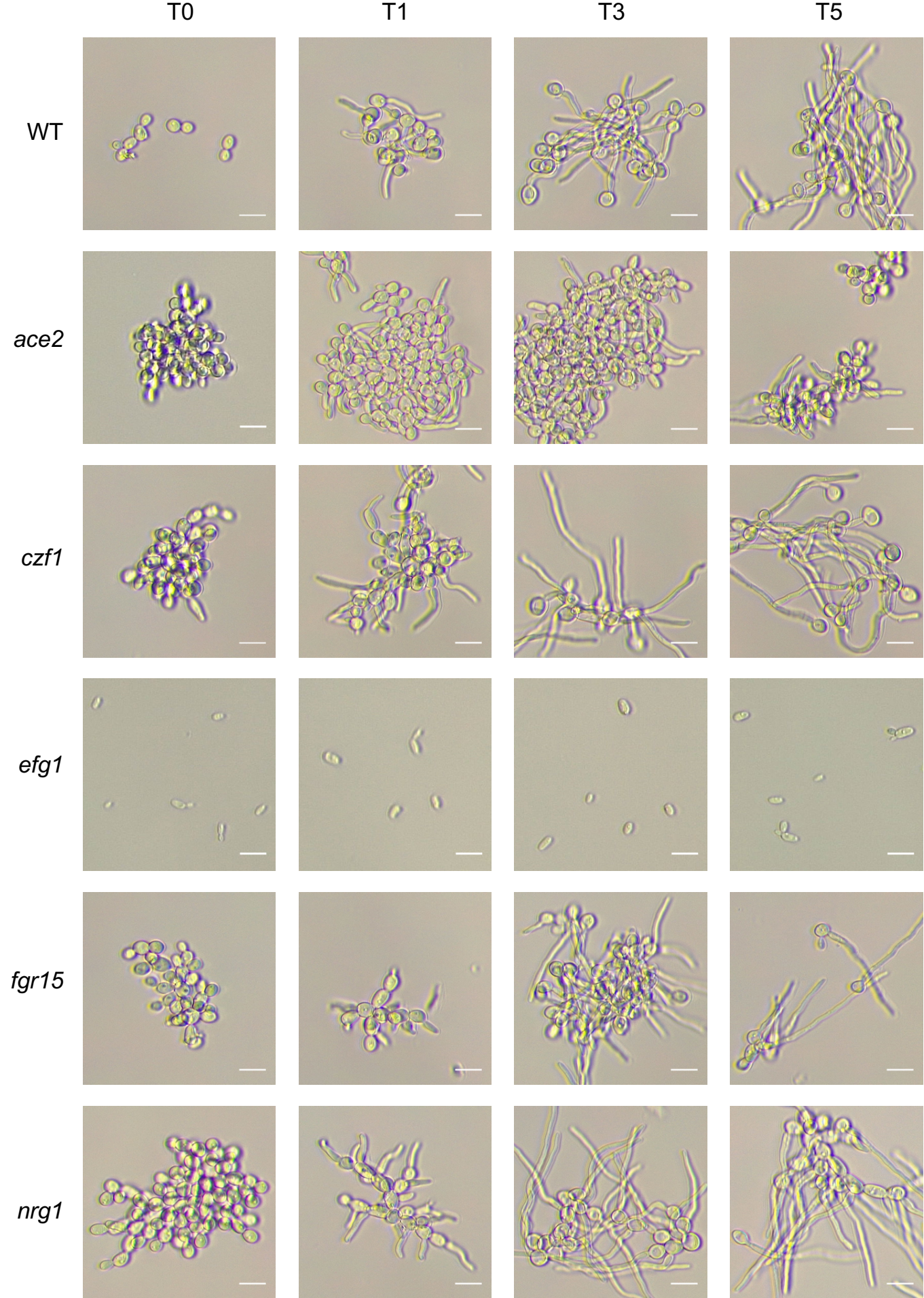

C. dubliniensis (2)

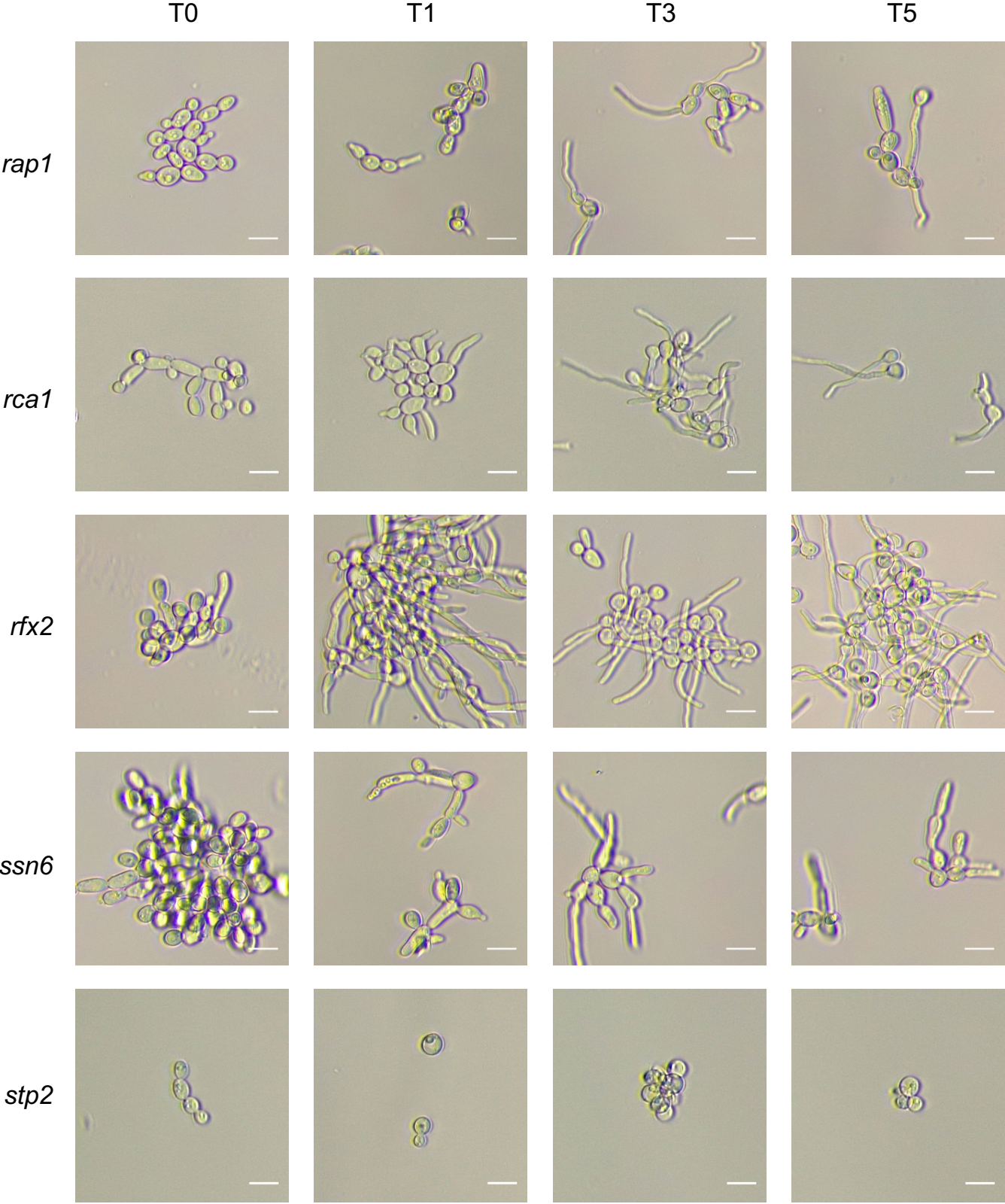

Supplement: Supplementary file 2 — Figure S2. Phenotypes of the mutants for which filamentation could not be quantified. Micrographs taken under an optical microscope at the time the cells were transferred to the filamentation inducing conditions (T0) and after one (T1), three (T3), and 5 h (T5) of filamentation. Only one of the C. dubliniensis isolates is shown although the phenotype was similar in the other isolate. The reference scale bars represent 10 μm. [file MMI-124-327-s001.pdf]

Supplementary Figure 3A

Time 0

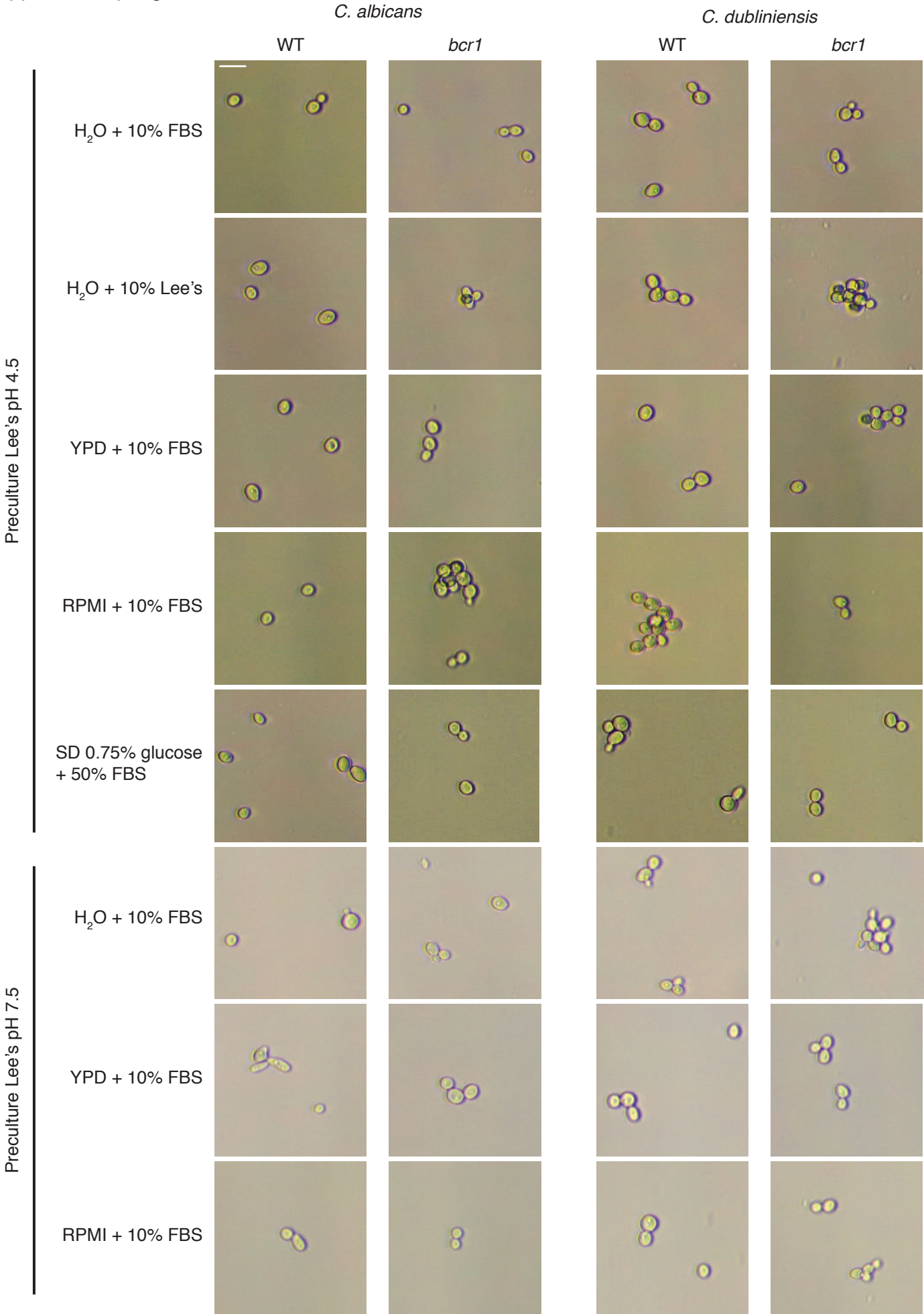

Supplementary Figure 3B

Time 1

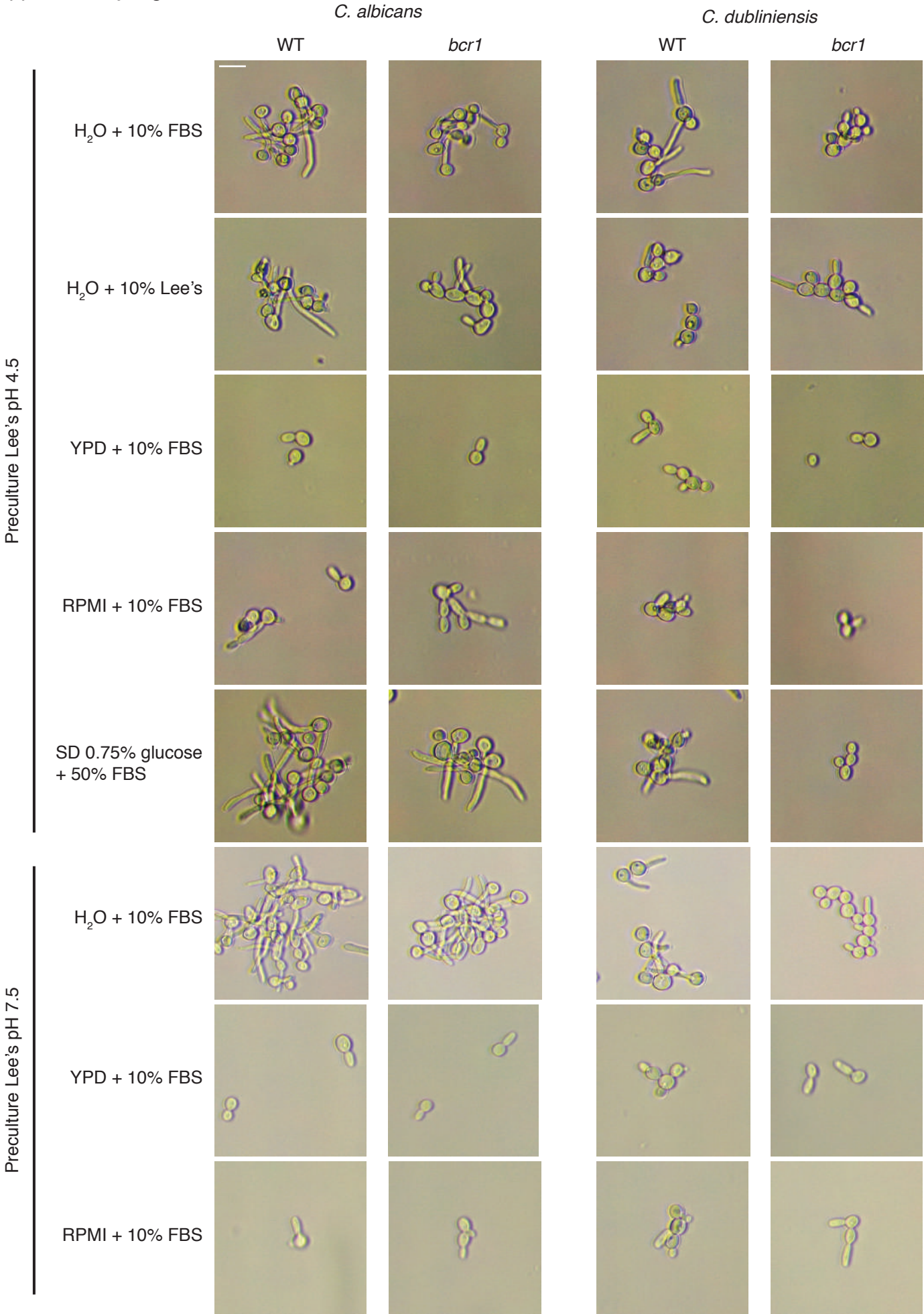

Supplementary Figure 3C

Time 3

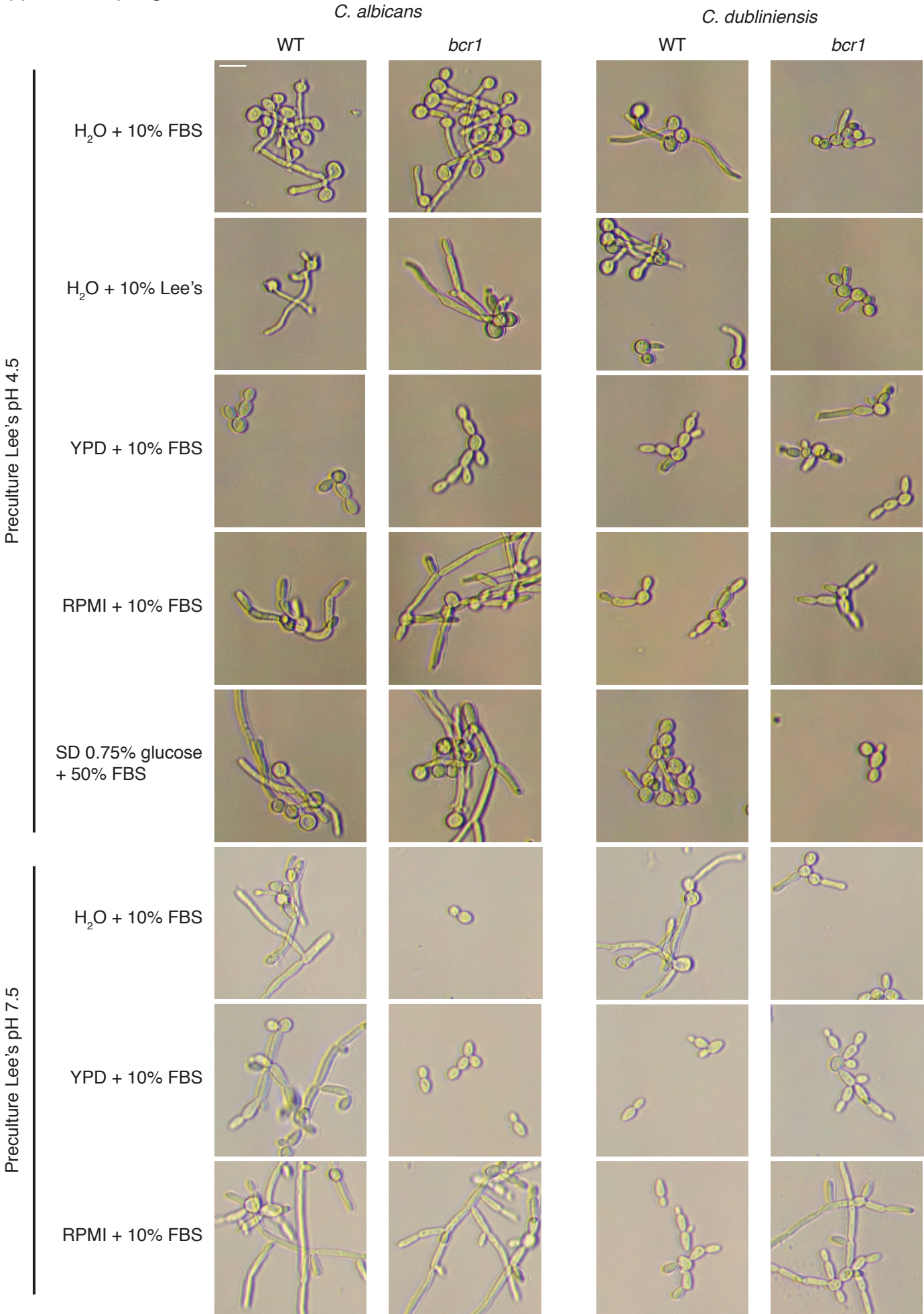

Supplement: Supplementary file 3 — Figure S3. Morphology of the bcr1 mutant under different filamentation inducing conditions. Micrographs under a light microscope of the reference strain and the bcr1 mutant of C. albicans and C. dubliniensis in the different media tested. As indicated at the furthest left, two preculture conditions were employed. Panel A, B, and C show micrographs taken at the 0, 1, and 3 h time points, respectively. The reference scale bar in the wildtype C. albicans in H20 + 10% FBS when preculture in Lee’s pH 4.5 was used represents 10 μm. [file MMI-124-327-s002.pdf]
