# Supplementary material for: Filamentation Profiling Reveals Multiple Transcription Regulators Contributing to the Differences Between Candida albicans and Candida dubliniensis
Source: Mol Microbiol. 2025 Jul 17;124(4):327–41. doi: 10.1111/mmi.70012 (PMC12510621; doi:10.1111/mmi.70012)

Supplementary Figure 4

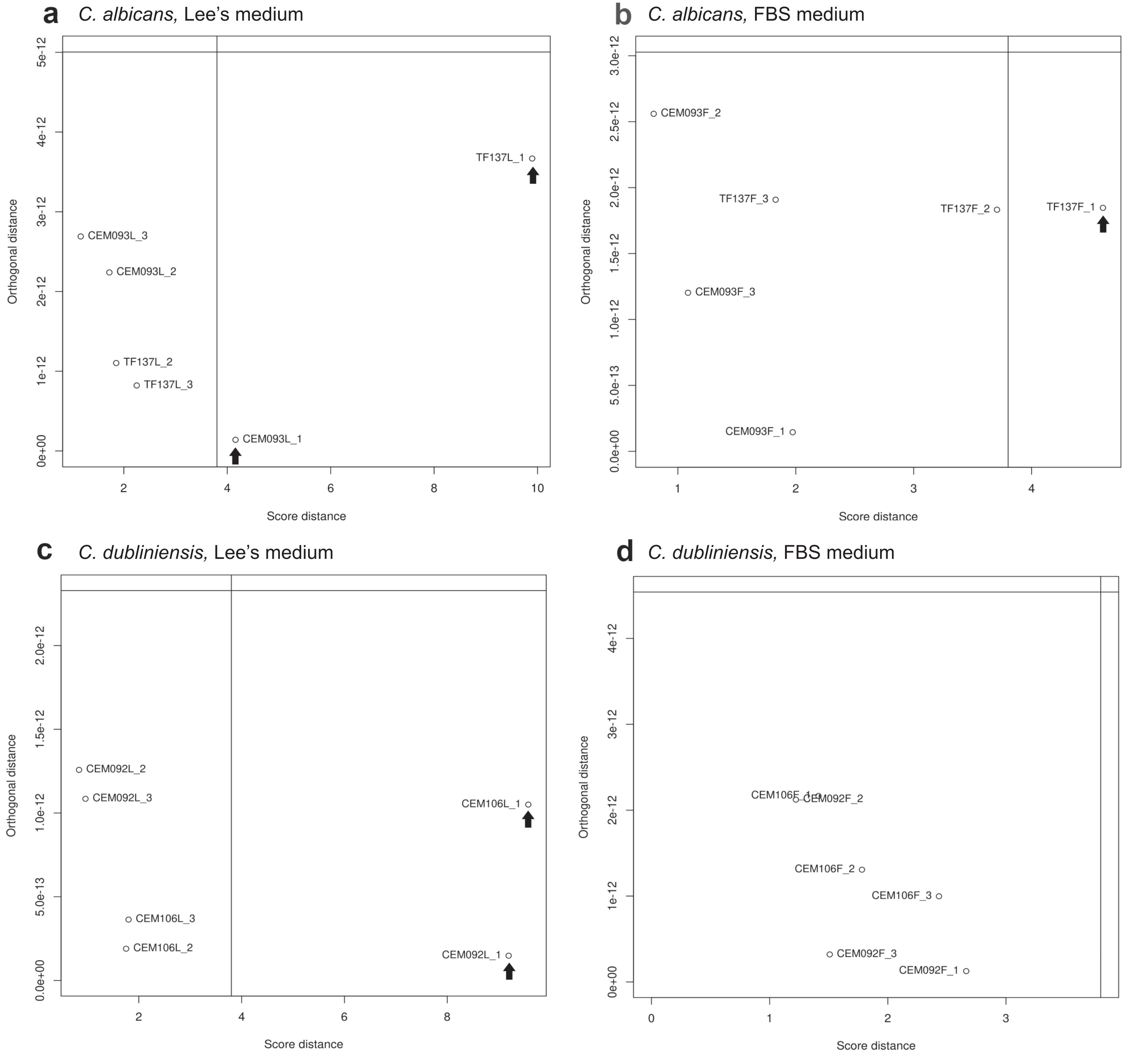

Supplement: Supplementary file 4 — Figure S4. Outlier map of the RNA‐seq replicates for the C. albicans (CEM093, WT strain and TF137, bcr1 mutant) in Lee’s (a) and FBS (b) media, as well as for C. dubliniensis (CEM092, WT strain and CEM106, bcr1 mutant) in Lee’s (c) and FBS (d) media. The black lines represent the cut‐off thresholds for orthogonal distances and the distance score (> 0.975). Replicates above any of the cut‐off thresholds were classified as outliers (indicated by a black arrow) and were excluded from subsequent analyses. [file MMI-124-327-s005.pdf]
